# Supported Binuclear Gold Phosphine Complexes as CO Oxidation Catalysts: Insights into the Formation of Surface‐Stabilized Au Particles

**DOI:** 10.1002/smsc.202400345

**Published:** 2024-10-14

**Authors:** Fabian Rang, Tim Delrieux, Florian Maurer, Franziska Flecken, Jan‐Dierk Grunwaldt, Schirin Hanf

**Affiliations:** ^1^ Institute for Inorganic Chemistry Karlsruhe Institute of Technology Engesserstr. 15 76131 Karlsruhe Germany; ^2^ Institute for Chemical Technology and Polymer Chemistry Karlsruhe Institute of Technology Engesserstr. 18/20 76131 Karlsruhe Germany; ^3^ Institute of Catalysis Research and Technology Karlsruhe Institute of Technology Hermann‐von‐Helmholtz‐Platz 1 76344 Eggenstein‐Leopoldshafen Germany

**Keywords:** CO oxidation catalysis, gold complexes, ligand effects, operando X‐ray absorption spectroscopies, phosphines, surface‐stabilized nanoparticles

## Abstract

Atomically precise gold phosphine complexes as precursors for supported Au catalysts tested in CO oxidation are presented. Using a variety of analytical techniques, including *in situ* and *operando* X‐ray absorption spectroscopy, it is discovered that minor changes in the ligand of the molecular complexes result in significantly different activation behaviors of supported Au catalysts under reaction conditions. When using [Au_2_(μ_2_‐POP)_2_]OTf_2_ (POP = tetraphenylphosphoxane) as single‐source precursor, an active supported oxidation catalyst in second light‐off is obtained, outperforming a commercial Au/TiO_2_ and a P‐free Au/Al_2_O_3_ reference catalyst. Conversely, using [Au_2_(μ_2_‐dppe)_2_]OTf_2_ (dppe = diphenylphosphinoethane) on alumina leads to a significant decrease in CO oxidation activity. This difference is attributed to the formation of P‐containing ligand residues on the support in the case of [Au_2_(μ_2_‐POP)_2_]OTf_2_/Al_2_O_3_, which enhances the thermal stability of the Au particles and affects the particle's electronic properties through charge transfer processes. This work provides insights into the dynamic ligand decomposition of molecular gold complexes under reaction conditions and demonstrates the delicate balance between the stabilization of Au particles, clusters, and complexes using ligands and the blocking of active sites. This knowledge will pave the way for the targeted use of molecular transition metal complexes as precursors in synthesizing surface‐stabilized nanoparticles.

## Graphical abstract

Atomically precise binuclear gold phosphine complexes used as precursors for supported Au catalysts in CO oxidation dynamically form highly active and stable surface‐stabilized nanoparticles with modulated electronic properties under reaction conditions.

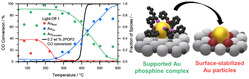

## 1 Introduction

Despite the fact that gold has been considered as a nonreactive metal for a long time, catalytic reactions driven by gold‐based catalysts have become a very hot topic in chemistry recently.^[^ ^1^ ^]^ Especially, in the field of heterogeneous catalysis, gold nanocatalysts have gained significant attention over the last decade.^[^ ^2^, ^3^ ^]^ The starting point for this development was laid by the investigations of Haruta and Hutchings, who both simultaneously postulated active heterogeneous gold catalysts for organic transformations.^[^ ^4^, ^5^ ^]^ Whereas Hutchings et al. investigated the vapor‐phase hydrochlorination of acetylene,^[^ ^6^ ^]^ Haruta and co‐workers reported the low‐temperature oxidation of carbon monoxide thanks to the use of small Au particles.^[^ ^7^ ^]^ Surprisingly, the CO oxidation reaction could already be carried out below 0 °C using supported ultrafine gold particles smaller than 10 nm in size as catalysts. Hereby, it was demonstrated that the choice of support material, such as α–Fe_2_O_3_, TiO_2_, Al_2_O_3_, ZrO_2_, MgAl_2_O_4_, and CeO_2_, has a tremendous impact on the catalytic activity.^[^ ^8^, ^9^, ^10^, ^11^, ^12^, ^13^ ^]^ Grunwaldt et al. have also shown that small gold colloids (≈2 nm) on TiO_2_ or ZrO_2_, prepared by reduction with tetrakis(hydroxymethyl)phosphonium chloride, are catalytically very active in the CO oxidation reaction.^[^ ^14^ ^]^ Rossi et al. extended this concept to the oxidation of organic molecules, such as alcohols, using molecular oxygen as oxidant under mild conditions.^[^ ^15^ ^]^

**Figure 1:**
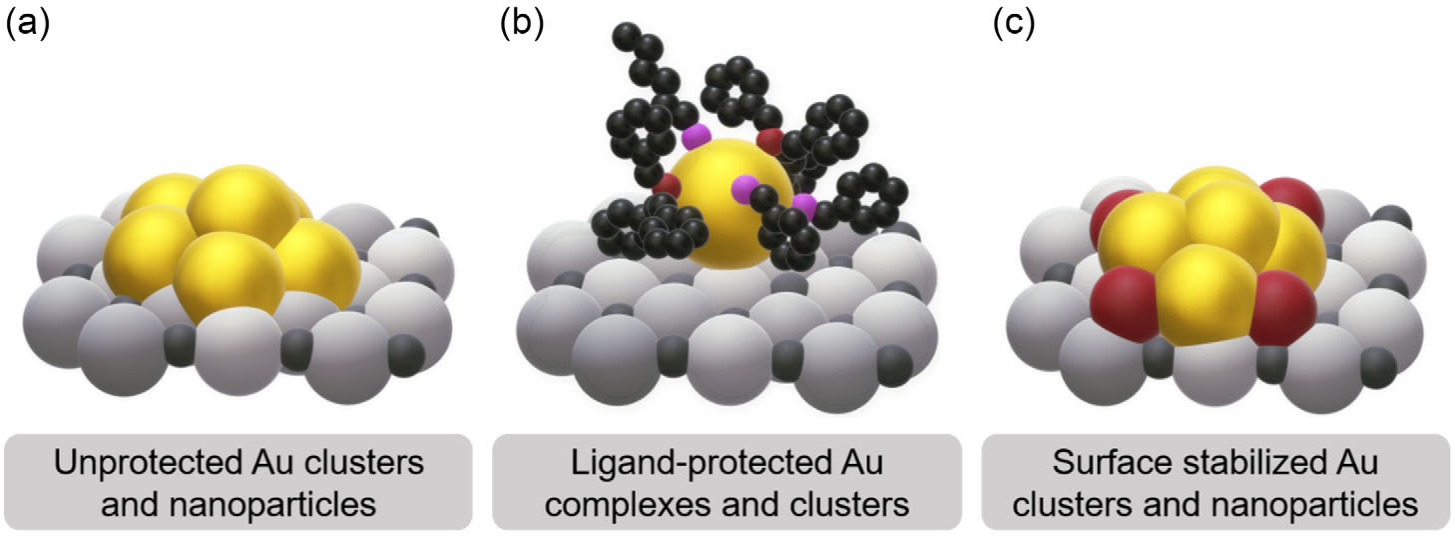
Schematic overview of a) unprotected Au clusters and nanoparticles, b) ligand‐protected Au complexes and clusters, and c) surface‐stabilized Au clusters and nanoparticles.

In addition to the support influence, the method of preparation, for example, impregnation or deposition precipitation, has proven to be crucial in controlling the activity of the catalysts.^[^ ^16^ ^]^ This complicates the reliable synthetic access to highly active Au oxidation catalysts and affects the robustness of catalytic test data.^[^ ^17^ ^]^ To overcome this challenge and guarantee the synthetic reproducibility, ligand‐protected Au nanoclusters have been investigated, which can be considered as nanobased model compounds toward larger Au particles.^[^ ^18^, ^19^, ^20^ ^]^ In contrast to Au nanoparticles, Au nanoclusters are monodisperse and exhibit well‐defined sizes and structures (**Figure** **1**a). The properties of such cluster compounds can be greatly influenced by a variety of parameters, such as the number of metal atoms,^[^ ^21^ ^]^ the coordination environment of the metal centers, and the choice of stabilizing ligands.^[^ ^22^ ^]^ Especially, the latter has found to be a versatile tool to tune the properties of noble metal complexes and clusters.^[^ ^23^ ^]^ In this context, thiols, carbenes, and phosphines, among other organic ligands, have found to not only stabilize metal clusters but also to modulate their electronic properties, thereby impacting their catalytic activity and selectivity (Figure 1b).^[^ ^24^ ^]^ Zhang et al. have, for example, shown that through the use of thiolate‐protected Au clusters, the chemoselective hydrogenation of the aldehyde groups of nitro benzaldehyde derivatives can be achieved, whereas deprotected Au clusters favored the hydrogenation of the corresponding nitro groups.^[^ ^25^ ^]^ However, the stabilizing ligands can also lead to the blocking of the active sites on the Au surface, which can cause severe decline in catalytic activity. Therefore the partial or complete removal of ligands has demonstrated to be important in order to create accessible Au sites and to guarantee sufficient contact between the surface metal atoms and the reactants.^[^ ^26^, ^27^, ^28^, ^29^ ^]^ For ligand removal, various activation strategies, such as thermal^[^ ^30^, ^31^ ^]^ and chemical treatments (oxidation and reduction),^[^ ^32^, ^33^ ^]^ as well as light‐induced approaches,^[^ ^34^ ^]^ have been investigated. However, the activation conditions must be carefully selected in order to counteract agglomeration and sintering processes, which often occur when the stabilizing ligands are being removed. Along these lines, also the stabilization of Au particles via ligand‐based residues and the formation of so‐called surface‐stabilized Au complexes and clusters have been investigated (Figure 1c).^[^ ^35^ ^]^ Hereby it has been shown that the decomposition of organic surfactants through carbonization at elevated temperatures can create a protective carbon shell around nanoparticles, which can counteract metal sintering.^[^ ^36^ ^]^ Also, Kempe et al. reported the use of a nickel salen complex as precursor for the formation of nickel particles stabilized by a nitrogen‐doped carbon shell on γ‐Al_2_O_3_ support.^[^ ^37^ ^]^ In the case of thiolate‐protected Au clusters, a sulfur ligand migration from Au to the support surface has also been reported and shown to be a decisive actor for tuning the catalytic activity.^[^ ^28^ ^]^ However, sulfur‐containing ligands are typically not considered for the preparation of active catalysts on a technical scale, due to their poisonous side effects.^[^ ^26^, ^38^ ^]^

Based on the fine balance between the stabilization of Au particles, clusters, and complexes by the use of ligands and the blocking of active surface sites through strongly coordinating molecules, we herein report a systematic study of the application of atomically precise binuclear gold phosphine complexes as precursors for supported Au catalysts using the oxidation of CO as test reaction. The focus was hereby to track the fate of the phosphine ligands before, during, and after the catalytic reactions *via* *in situ* and *operando* spectroscopic techniques.

## 2 Results and Discussion

### 2.1 Catalyst Synthesis and Characterization

**Figure 2:**
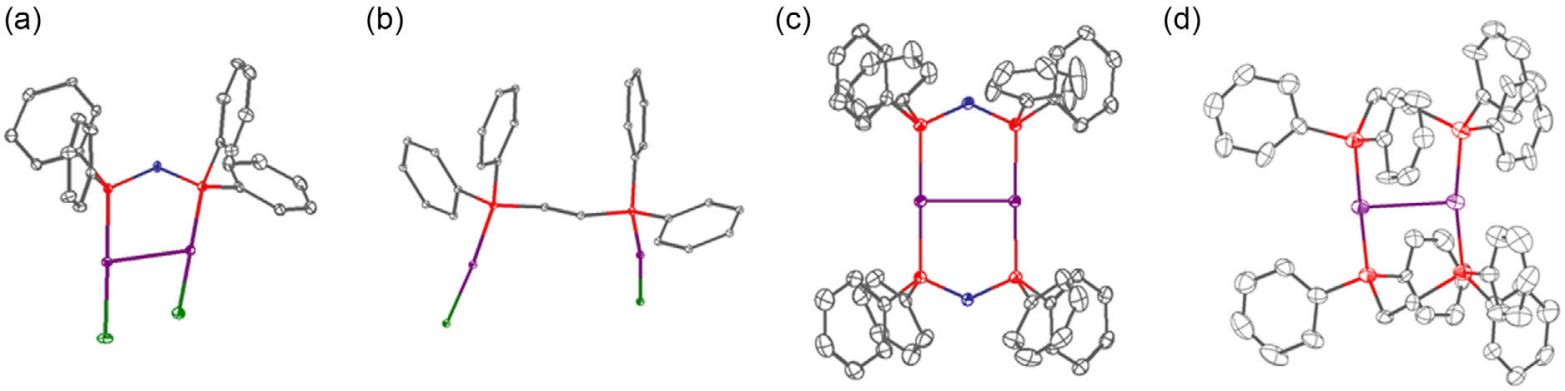
Overview of the crystal structures of a) [Au_2_(μ_2_‐POP)_2_Cl_2_] and b) [Au_2_(μ_2_‐dppe)Cl_2_] and the cations of c) [Au_2_(μ_2_‐POP)_2_]OTf_2_ and d) [Au_2_(μ_2_‐dppe_2_]OTf_2_. Hydrogen atoms, counter anions, and solvent molecules are omitted for clarity. Ellipsoids drawn at the 30% probability level. Gray: carbon; blue: oxygen; red: phosphorous; green: chlorine; and purple: gold.

To study the effects of the choice of molecular gold precursor for the synthesis of heterogeneous Au‐based nanocatalysts on the catalyst activity and stability, the ligands tetraphenyldiphosphoxane (POP) and 1,2‐bis(diphenylphosphino)ethane (dppe) were used to synthesize dinuclear gold complexes with one or two chelating phosphine ligands. Dinuclear complexes were selected, since they often show superior stability in comparison to mononuclear Au complexes. These highly defined molecular complexes (**Figure** **2**) were then used as starting point for the synthesis of heterogeneous catalysts via incipient wetness impregnation (IWI) onto y‐Al_2_O_3_ support with 0.2 wt% Au loading. While alumina is generally outperformed by reducible supports, such as TiO_2_ or Fe_2_O_3_, during CO oxidation because of stronger metal‐support interactions,^[^ ^7^ ^]^ alumina was specifically chosen to minimize the influence of support interactions on the catalyst's activity during CO oxidation reactions. The resulting catalysts are abbreviated by the number of gold atoms within the complex and the type and amount of ligands present, for example, the catalyst made based on μ_2_‐1,2‐bis(diphenylphosphino)ethane‐bis{chlorido‐gold(I)} ([Au_2_(μ_2_‐dppe)_2_Cl_2_]) on alumina is referred to as 2dppeCl. In addition to the supported gold phosphine complexes, two reference catalyst were investigated. One reference catalyst was synthesized via IWI of chloroauric acid onto the alumina support (AuRef). This system serves to highlight the effect of the ligands, as no additional phosphine is present with the precursor. IWI as synthesis methods was specifically chosen at this point to retain the preparation method to the before‐described impregnated Au complexes. In addition, commercially available Au on TiO_2_ (Au/TiO_2_), commonly used as a CO oxidation catalyst in the literature, was utilized as a reference catalyst.^[^ ^39^, ^40^, ^41^, ^42^ ^]^ The gold loading on the support was verified by inductively coupled plasma–optical emission spectroscopy (ICP–OES) measurements and the specific surface area, determined by the Brunauer–Emmett–Teller method, and was shown to be only minorly influenced by gold loading compared to the Al_2_O_3_ support material (Table S2, Supporting Information).

Attenuated total reflection‐infrared (ATR‐IR) spectroscopy was performed to confirm the successful impregnation of the Au complexes in the as‐prepared state and after a short thermal treatment in air at 600 °C for 10 mins (Figure S2–S5, Supporting Information). For this, the spectra of supported and molecular Au complexes, with the strongest vibrations of the respective complexes being the aromatic C—H deformation vibrations around 690 and 1105 cm^−1^ and the aromatic C—C stretching bands at 1435 cm^−1^, were compared to verify the presence the gold complexes on the support material. Based on the IR spectra, the successful impregnation of the complexes onto the Al_2_O_3_ support was proven. However, as seen in the ATR‐IR spectrum of the 2POPCl catalyst (Figure S5, Supporting Information), while the prominent aromatic vibrations can be observed, the characteristic P—O vibration at 910 cm^−1^ of the POP‐based Au complex^[^ ^43^ ^]^ is not visible any more after the impregnation step. This points to the fact that the POP ligand decomposes during the impregnation process as it is known to be air sensitive. After thermal treatment of all supported complexes at 600 °C, solely the vibrations of the alumina support are visible, correlating to the (partial) decomposition of the organic ligands on the surface, yet the IR results do not give further information about the presence of ligand residues on the support surface.

**Figure 3:**
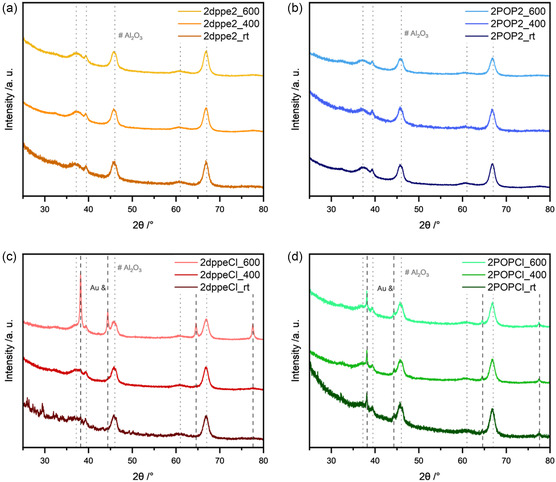
Powder X‐ray diffractograms of a) 2dppe2, b) 2POP2, c) 2dppeCl, and d) 2POPCl after the impregnation and after thermal treatment at different temperatures (400 and 600 °C) for 10 min. rt = room temperature. (COD ID: Al_2_O_3_: 1 010 461 Au: 9 013 035).

To evaluate the stability of the supported Au complexes further, powder X‐ray diffraction (PXRD) analyses were employed. The diffractograms of the impregnated Au complexes, shown in **Figure** **3**, indicate no reflexes assigned to the Au lattice plane for any of the supported Au complexes, indicating the absence of larger (>10 nm) Au nanoparticles. The only exception is 2POPCl, where sharp reflexes at 38.1°, 44.3°, 64.7°, and 77.5° were observed, which point toward the formation of larger Au particles of about 18 nm (based on the Scherrer equation) on the support and underline that the initial Au complex decomposed during impregnation, which consequently leads to the sintering and the formation of larger Au crystallites. This corroborates with the results obtained from ATR‐IR spectroscopy, whereby the absence of the characteristic P—O stretching frequency already indicated the decomposition of the POP ligand. Interestingly, 2POPCl is also the only catalyst that has a different color after impregnation, displaying a slight blueish hew, while the other catalysts remain white. Additional PXRD measurements after a thermal treatment at 400 and 600 °C in air for 10 min were conducted to determine the point of decomposition of the supported complexes and to follow the sintering behavior (Figure 3). While a treatment at 400 °C did not lead to any further sintering of the supported Au complexes, 2dppeCl displayed Au‐related reflexes, corresponding to crystallites of around 24.4 nm, after the thermal treatment at 600 °C. Both of the catalysts, which possess two phosphine ligands surrounding the gold atoms, 2dppe2 and 2POP2, continued to show no signs of sintering even after the thermal treatment at 600 °C. This hints toward a strong thermal stability of the gold species on the surface of the support, which might be attributed to an interaction between remaining phosphorus‐containing residues and Au nanoparticles. This interaction leads to an increase of the stability of the Au atoms toward thermally induced sintering processes. A similar behavior was observed by the group of Kempe when examining a nickel salen precursor complex for the reductive amination of ketones by ammonia and hydrogen. After thermal treatment of the nickel salen complex on γ‐Al_2_O_3_, the thereof resulting nickel nanoparticles were surrounded by a nitrogen doped carbon shell, which aided in stabilizing the metal centers and hindering sintering during the reductive amination.^[^ ^37^ ^]^

To gain further insights into the thermal stability of the catalysts, which is a crucial factor for the formation of a binding pocket for Au species as reported previously by Zhang et al. in the context of thiolate‐protected gold clusters,^[^ ^28^ ^]^ thermogravimetric analyses coupled with mass spectrometry were conducted. The results are depicted in the ESI (Figure S6–S9, Supporting Information) and show a varying degree of ligand decomposition as well as different decomposition temperatures and pathways. It was found that the order of combustion temperatures (2dppe2 > 2POP2 > 2dppeCl > 2POPCl) determined via thermogravimetry and mass spectrometry (TG‐MS) aligns with the trend observed during the thermal decomposition of the molecular complexes, as shown in Table S1, Supporting Information.

### 2.2 Catalytic CO Oxidation Activity of Supported Au Complexes

**Figure 4:**
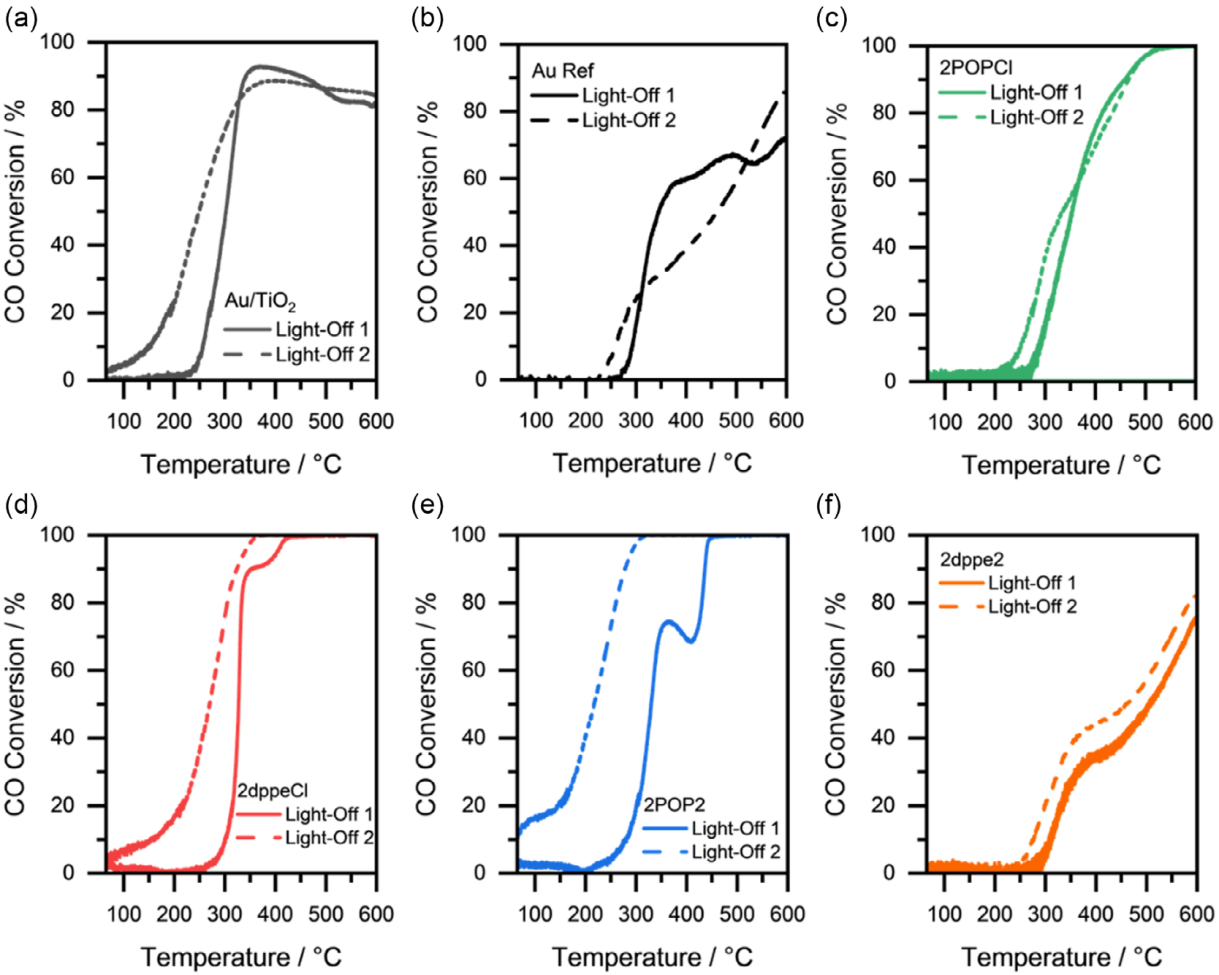
Catalytic activity of tested samples for a gas mixture of 1000 ppm CO, 10 vol% O_2_ in N_2_. a–f) The two‐consecutive light‐off curves for a) Au/TiO_2_, b) AuRef, c) 2POPCl, d) 2dppeCl, e) 2POP2, and f) 2dppe2.

The catalytic properties of the supported Au complexes were probed by CO oxidation tests performed under transient (during heat up) and more applied conditions (at high weight hourly space velocity [WHSV] and temperatures), which are known to cause severe deactivation due to sintering. Two consecutive light‐off (LO)/light‐out cycles (light‐off corresponds to the heating step, whereas light‐out describes the consecutive cooling step) were performed in order to evaluate reversible and irreversible changes during the first LO, which can also be seen as a pretreatment step. Harsher conditions were mimicked at a high WHSV of 60 000 L gAu^−1^ h^−1^ with temperatures up to 600 °C. This temperature regime was selected, since IR and PXRD studies have shown that at these temperatures, the sintering and the formation of Au nanoparticles occur. Due to the high flow rate, we expect that carbonization of the supported catalysts can be prevented, considering earlier studies by Kempe et al.^[^ ^37^ ^]^ As mentioned before, a commercial Au/TiO_2_ catalyst^[^ ^44^, ^45^ ^]^ and an additional phosphine‐free Au reference catalyst on alumina (AuRef) were used as benchmark examples. The activity of the different catalysts will be compared based on T_10_, T_50_, and T_90_, the temperatures at 10%, 50%, and 90% CO conversion, respectively, with the results being shown in **Table** **1** and **Figure** **4**.

**Table 1:** Temperatures at 10%, 50%, and 90% CO conversion during light‐off 1 (columns 2–4) and 2 (columns 5–7).

	T_10,1_ [°C]	T_50,1_ [°C]	T_90,1_ [°C]	T_10,2_ [°C]	T_50,2_ [°C]	T_90,2_ [°C]
2dppeCl	305	330	340	150	270	300
2dppe2	310	510	–	290	350	–
2POPCl	290	330	460	260	320	460
2POP2	280	310	440	< 50	220	280
AuRef	290	345	–	250	450	600
Au/TiO_2_	260	300	340	150	240	–

From the results it becomes obvious that during the first light‐off, the reaction onset already largely depends on the choice of ligand within the molecular Au phosphine precursor. While with the POP‐based catalysts, 2POP2 and 2POPCl, the CO oxidation reaction starts to occurs around 280 °C, with T_10,1_ values of 280 and 290 °C, respectively, the T_10,1_ values of the dppe‐based catalysts are noticeably higher, at 305 °C for 2ppeCl and 310 °C for 2dppe2. For the second light‐off, all supported Au complexes show a strong improvement in catalytic activity with a much earlier reaction onset. The best performing catalyst 2POP2 with T_10,2_ < 50 °C, T_50,2_ = 220 °C, and T_90,2_ = 280 °C outperforms the commercial reference catalyst Au/TiO_2_ in terms of T_50,2_ by 20 K and the phosphine‐free reference catalyst AuRef even by 230 K. This is interesting as usually Au/TiO_2_ catalysts are reported to be more active than Au/Al_2_O_3_ counterparts.^[^ ^10^ ^]^ The behavior points toward nonreversible structural changes during the first light‐off, which are differently pronounced depending on the ligand present in the molecular precursor. The first light‐off can therefore also be considered as crucial pretreatment for the activation of the supported Au complexes. In the case of 2POP2, the experimental data points toward a partial ligand decomposition under reaction conditions followed by the formation of catalytically active surface‐stabilized Au species. Additionally, to the improvement in the low‐temperature regime, no decrease of the activity at higher temperatures is observed for 2POP2 in contrast to the commercial Au/TiO_2_ catalyst, where in the second light‐off, a maximum of 87% CO conversion is reached at 400 °C, which again diminishes up to 600 °C. Interestingly, the catalyst based on two dppe ligands, namely 2dppe2, shows a completely different behavior, with an onset in CO oxidation activity at around 300 °C corresponding to a T_10,1_ of 310 °C with a rather flat increase in CO conversion leading to a T_50_,1 of 510 °C and a maximum of 75% CO conversion at 600 °C. Despite the fact, that the second light‐off leads to a slight improvement with respect to the T_10,2_ and T_50,2_ values, the shape of the curve stays similar to the first LO. The generally low catalytic activity of 2dppe2 can be attributed to the very high stability of the molecular precursor, as demonstrated by temperature‐dependent PXRD and TG‐MS measurements, which might lead to a blocking of the active Au sites and a diminished catalytic activity. Similar observations were made by Truttmann et al. who studied the influence of thiolate and phosphine ligands on the catalytic activity of ceria‐supported Au nanoclusters in the oxidation of CO.^[^ ^29^, ^31^ ^]^

To unravel the influence of the different ligands, their fate, and structural transformation during the synthesis as well as during the CO oxidation catalysis, a plethora of analytic techniques was employed, such as transmission electron microscopy (TEM) analysis before and after the light‐off cycles, *operando* X‐ray absorption spectroscopy (XAS), and diffuse reflectance infrared Fourier transformed spectroscopy (DRIFTS) using CO as probe molecule. This is not only crucial to understand the dynamic structural changes that are occurring when using molecular phosphine Au complexes as precursors for the synthesis of highly active heterogeneous CO oxidation catalysts, but also to investigate the impact of ligands and ligand‐based residues on the electronic properties of the metals, as shown to be an important factor by Sufyan et al.^[^ ^46^ ^]^

### 2.3 Characterization of the As‐Prepared and Spent Catalysts

**Figure 5:**
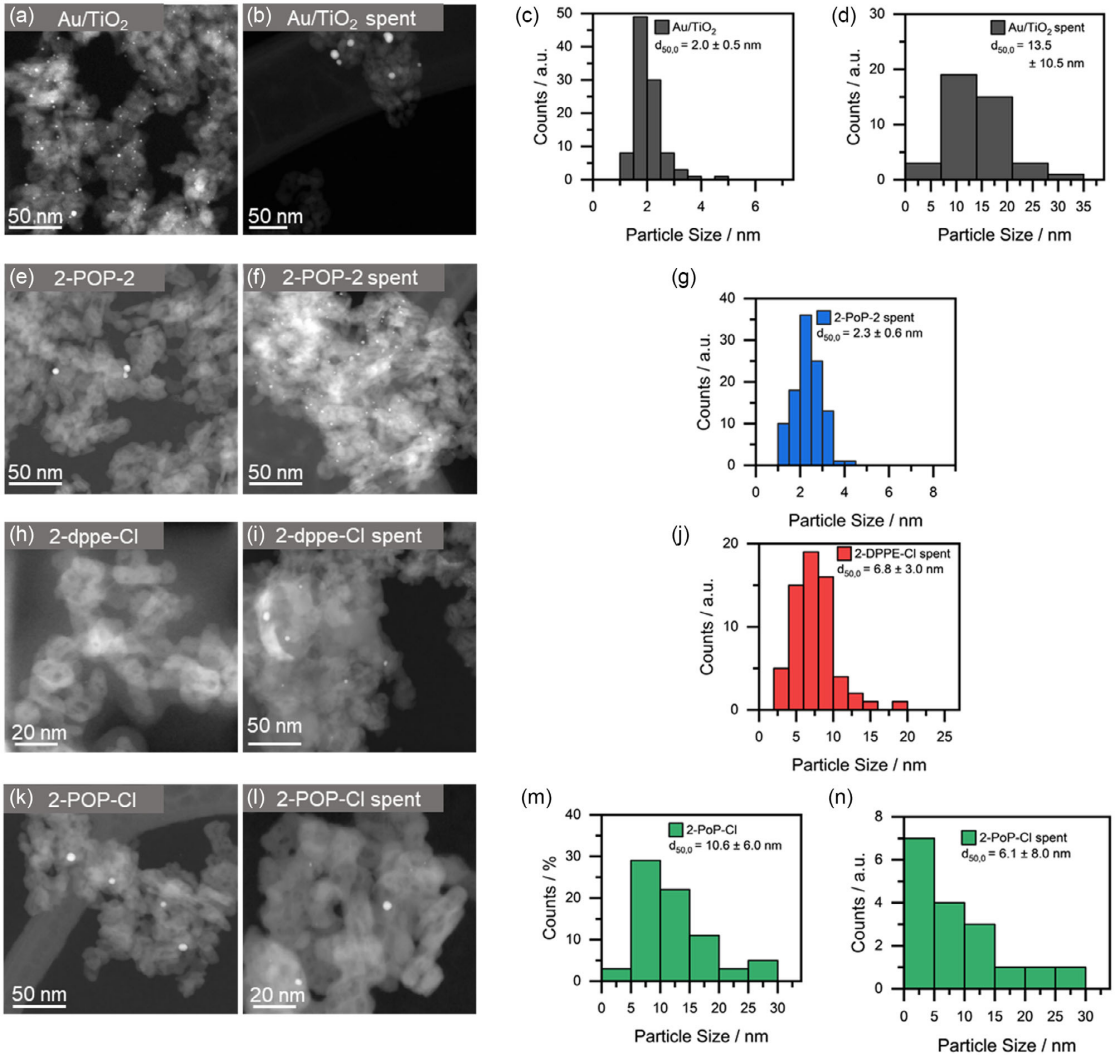
TEM images of the different a,b,e,f,h,i,k,l) Au catalysts and the corresponding particle size distributions c,d,g,j,m,n) before and after the reaction.

It is well known in literature that the size of nanoparticles is one of the most decisive points to achieve high catalytic activity for Au‐based catalysts.^[^ ^47^, ^48^, ^49^, ^50^ ^]^ This is due to the fact that size and electronic state of the supported metal particles are strongly linked. Therefore, TEM analyses of selected catalyst candidates were conducted to determine the Au particle sizes of the catalysts before and after the light‐off cycles (**Figure** **5**).

In the case of 2POP2, no Au particles are found within the fresh catalyst with the exception of a few smaller particles, emphasizing the high degree of stabilization provided by the ligand. After the light‐off cycles, particles with a size of 2.3 ± 0.6 nm are formed, which are narrowly distributed, further underlining the stabilizing effect of the remnant ligands even during the applied reaction conditions. The small particles that are formed are also in line with the increased activity for the second light‐off as they continue to lie within the deemed optimal particle size for Au nanoparticles for the CO oxidation reaction.^[^ ^49^ ^]^ It is also of note that the size resembles the initial particle size of the commercial catalyst, which initially showed the highest catalytic activity. Similar as in the case of 2POP2, 2dppeCl does not show particles in the initial state of the freshly prepared supported Au catalyst, indicating a stabilization effect of the ligand. However, after the light‐off cycles, particles with 6.8 ± 3.0 nm are formed, which is in line with the decline in catalytic activity. In contrast, 2POPCl consists of initially large particles with a median size of 10.6 ± 6.0 nm, which was already shown by PXRD analyses of the freshly impregnated Au complex. After the light‐off cycles, particles with a size of 6.1 ± 8.0 nm are found. Note that only a few particles were found in the images as indicated by the lower count rate and therefore the reduction in particle size should not be overstressed. However, the TEM images of the 2POPCl catalysts indicate that the ligands did not stabilize the Au atoms, thus forming large particles during the impregnation, which agree well with the PXRD measurements and again correlate with the observed lower catalytic activity.

To compare the within this work prepared on supported Au complexes with an established Au catalyst, the commercial Au/TiO_2_ catalyst was investigated before and after the catalytic reaction using TEM imaging. Hereby an initial narrow distribution of finely dispersed particles and a median particle size of 2.0 ± 0.5 nm was observed (Figure 5a). Under reaction conditions the particle size then increases, forming particles with a size of 13.5 ± 10.6 nm, as shown in Figure 5b. This indicates sintering of the Au species on the support during operation, which is also validated by the lower number of visible particles in the image shown.

The before and after TEM images have clearly shown that the particle size is a crucial factor for the corresponding catalytic activity. Whereas the commercial Au/TiO_2_ catalyst initially displays the smallest particle size and the highest catalytic activity, 2POP2 ends up with the highest activity and smallest particle size, which is similar to the initial state of the commercial catalyst. This trend is further corroborated by the results obtained for the 2dppeCl and 2POPCl catalysts, which both contain larger particles and consequently show a diminished catalytic activity when compared to 2POP2. Based on the catalytic study and the TEM analyses, the choice of ligand has shown to be very important in the context of the sintering resistance. Consequently, 2POP2, 2POPCl, and 2dppeCl were further examined by means of *operando* methods, with the aim to uncover the influence and fate of the ligands during the CO oxidation reaction in more detail.

### 2.4 The Fate of Supported Au Complexes as Catalysts under Lean CO Oxidation Conditions

**Figure 6:**
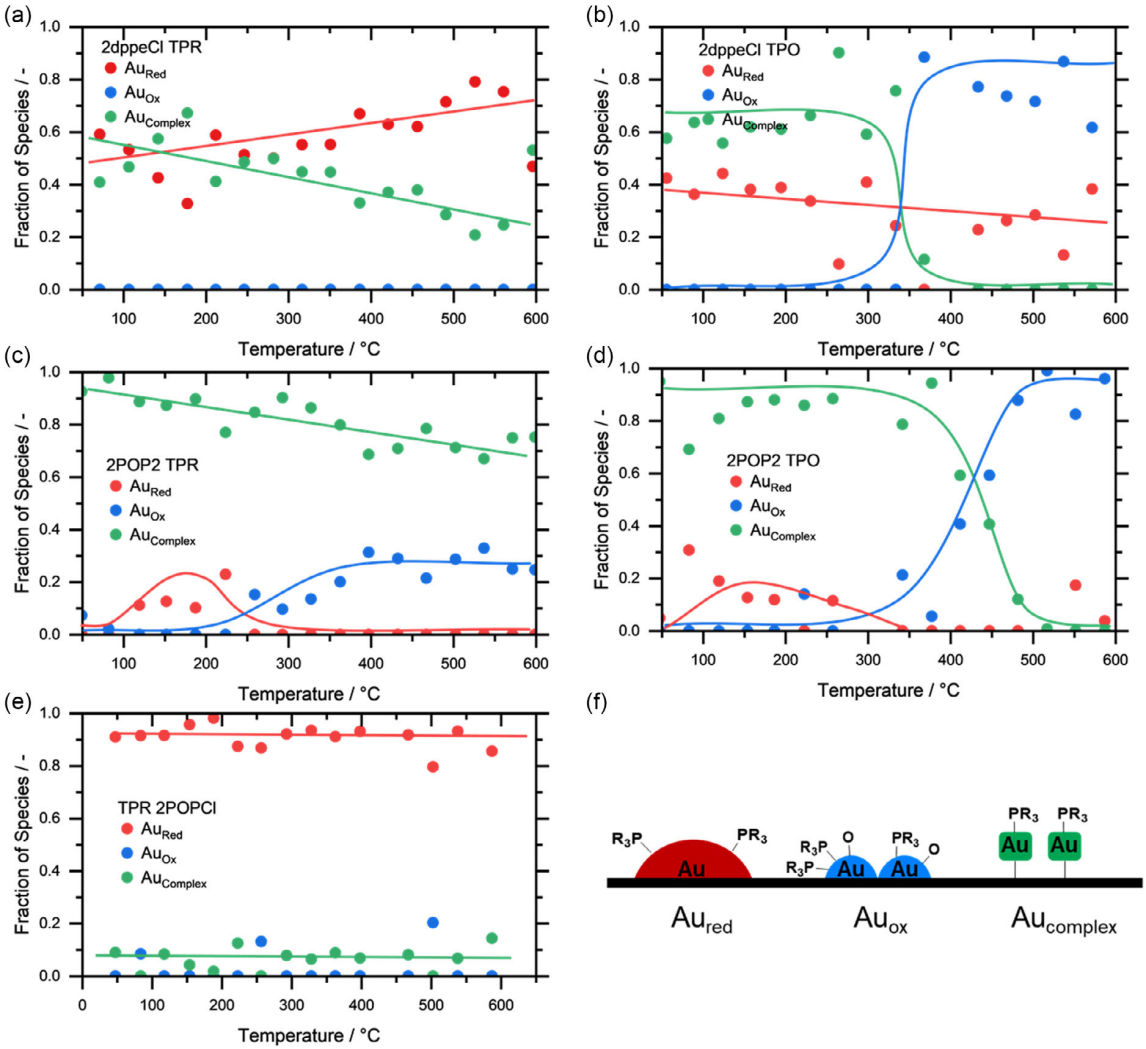
*In situ* TPR and TPO measurements for 2dppeCl, 2POP2, and 2POPCl. The measurements show the evolution of potential Au species determined by MCR‐ALS for 2dppeCl, 2POP2, and 2POPCl a), c), and e) in reducing conditions and b) and d) in oxidizing conditions, respectively. Solid lines are given as guide for the eye. f) Schematic visualization of the three species determined by MCR‐ALS.

Noble metal catalysts are highly dynamic systems, which undergo significant changes during operation depending on the initial particle size or the use of any additives.^[^ ^51^ ^]^ As discussed in the previous sections, the binuclear gold phosphine complexes (partially) decompose at different temperatures, possibly leading to distinct structural changes and consequently to different catalytic activities. Even though the reaction mixture contains only two reactive species, CO and O_2_, it is challenging to deconvolute their influence on the structural changes of heterogeneous catalysts during catalysis. Therefore, in the first step, *in situ* XAS experiments were performed under oxidizing and reducing conditions. Those experiments help to investigate the oxidation state and particle size of the Au catalyst under 10% O_2_ or 1000 ppm CO atmosphere. The obtained spectra were fit with the spectra gained by the multivariate curve resolution‐alternating least square (MCR‐ALS) method. MCR‐ALS allows to recover pure spectra of chemical compounds in an unresolved mixture, when no prior information is available.^[^ ^52^ ^]^ Here, it should be noted that the MCR‐ALS analysis is based on an algorithm and provides a mathematical approximation of real data. This strategy was pursued since no Au reference spectra represent sufficiently the molecular Au phosphine complexes. For example Maurer and Ankudinov et al. have recently shown that bulk reference spectra do not match with the spectra of their corresponding molecular complexes.^[^ ^53^, ^54^ ^]^ In addition, we observed that the spectra of the molecular Au complexes changed after the impregnation step; hence, they are not suited as possible reference points. To perform the MCR‐ALS analysis, all obtained spectra were considered and analyzed with a principal component analysis (PCA) first. The PCA resulted in a total of three pure components, which represent the dataset. The corresponding extracted spectra from MCR‐ALS (Figure S10, Supporting Information) were assigned based on the experimental spectra: a species, which corresponds to reduced gold (Au_Red_, red), partially oxidized gold (Au_Ox_, blue), and the precursor gold complexes (Au_Complex_, green), all of which are depicted in **Figure** **6**. Note that the graphical representations of the assigned species, shown in Figure 6f, are simplified, but show the different electronic character of the assigned species, induced by the presence of additional ligand residues. The spectra of the supported Au complexes correspond to typical Au(I)‐spectra, as indicated by the reference spectra of typical Au(I) precursors (Figure S11, Supporting Information).

Figure 6a shows that 2dppeCl consists of a mixture of Au_Red_ and Au_Complex_ species initially. In the presence of 1000 ppm CO in He and at ≈260 °C the amount of Au_Red_ starts to slightly increase up to 510 °C, where a maximum of Au_Red_ species is reached making up roughly 80% of the species in the catalyst, while 20% are represented by Au_Complex_. With increasing temperature this effect seems to be reversed. Under oxidizing conditions, a change in composition at 300 °C can be witnessed, which is in line with the offset of catalytic activity. Upon reaching the final temperature of roughly 600 °C, the catalyst consists of 60% Au_Ox_ and 40% Au_Red_, which could indicate a sintering of the catalyst at elevated temperatures. The sintering at higher temperatures and the formation of Au crystallites (24.4 nm) was also shown by PXRD, which can be seen in Figure 3.

2POP2 initially almost consists entirely of Au_Complex_, as shown in Figure 6c. This is in line with the PXRD analysis of the fresh catalyst, where no Au crystallites were observed. Under reducing conditions, Au_Ox_ is formed, which can most likely be explained by a partial decomposition of the ligands and corresponding complexes due to higher temperatures, as previously observed during TG‐MS measurements (Figure S7, Supporting Information). Remaining phosphorus‐based species on the surface of the support enable a charge transfer from Au to P, which explains the formation of Au^δ+^ species. In oxidizing conditions, it can be observed that around 200 °C the catalyst starts to form Au_Ox_, which is very well in line with the onset in catalytic activity as observed in the light‐off cycles. Furthermore, the increase of the Au_Ox_ species does not take place as rapidly, when compared to 2dppeCl, which also resembles the slow increase in catalytic activity during the first cycle. By the end of the experiment the catalyst is almost exclusively made out of Au_Ox_, with some minor amounts of Au_Red_ suggesting no strong degree of sintering, which is supported by the TEM images shown in Figure 5f, where only small particles of 2.3 ± 0.6 nm were observed after the light‐off cycles.

Finally, in contrast to 2POP2, the 2POPCl catalyst starts in a fully reduced state, which does not change under reducing conditions. This is in line the previous characterizations via TEM imaging (Figure 5), PXRD (Figure 3), and ATR‐IR (Figure S6, Supporting Information), which all displayed signs of the POP ligand decomposition and Au crystallite formation on the surface of the support straight away after impregnation.

**Figure 7:**
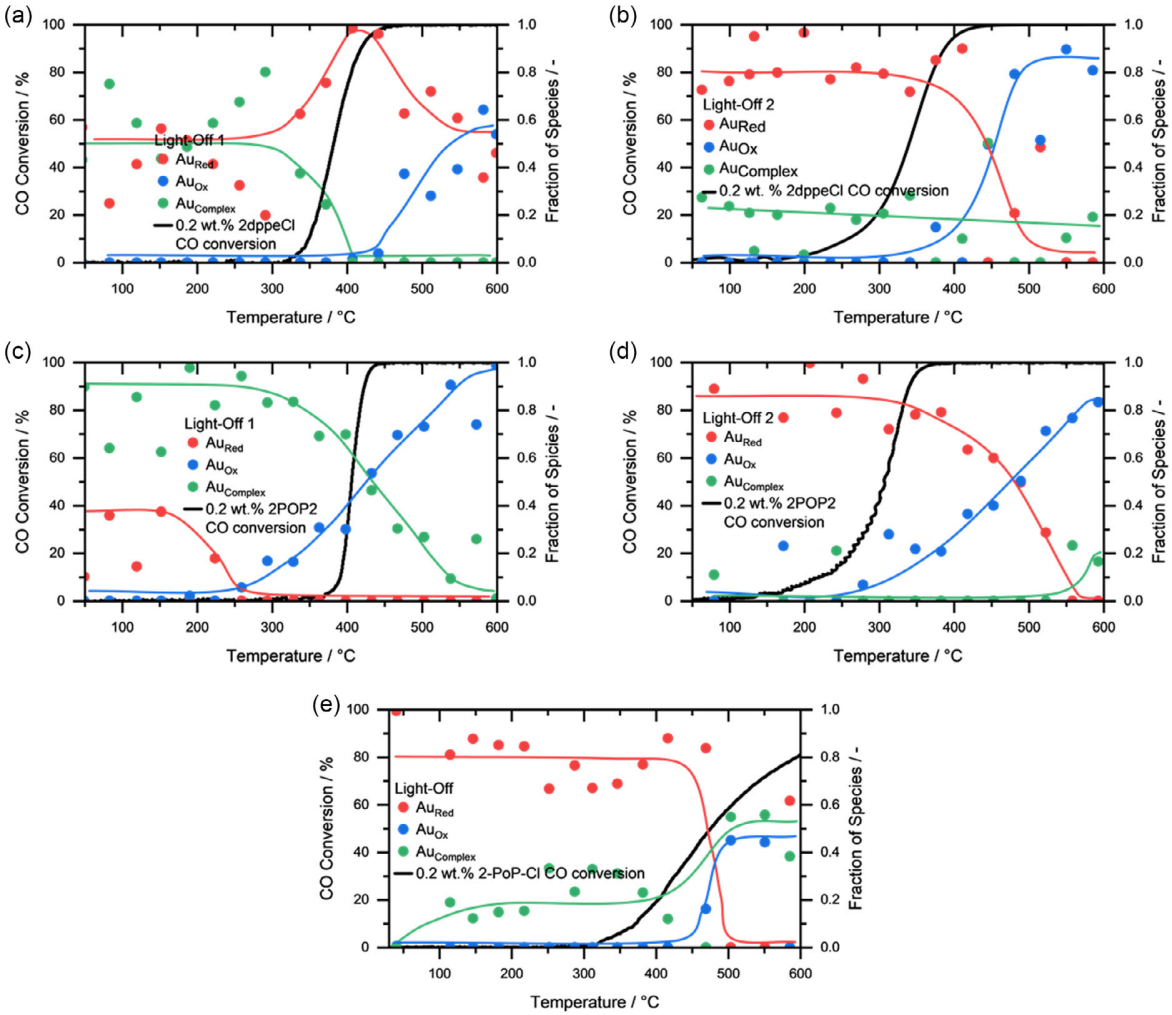
Transient light‐offs (1000 ppm CO, 10% O_2_ in He, WHSV: 170 000 L gAu^−1^ h^−1^) for the three samples 2dppeCl, 2POP2, and 2POPCl. The oxidation states during CO conversion with a) light‐off 1 and b) light‐off 2 for 2dppeCl, c) light‐off 1, d) light‐off 2 for 2POP2, and e) light‐off 1 for 2POPCl were derived by linear combination fitting of *operando* XANES data.

The conducted *in situ* XAS measurements provide insight into the stability of the supported Au complexes under oxidizing and reducing conditions and showed that the ligand decomposition can lead to P‐containing residues on the support, which alter the electronic structure of the metal centers. Under oxidizing conditions and elevated temperatures 2dppeCl started to sinter to a greater degree than 2POP2, which explains the enhanced catalytic activity during the second light‐off cycle of 2POP2. However, the measurements do not provide any insights into the dynamic changes, dependent on the choice of stabilizing ligands, and consequently *operando* XAS measurements were carried out. **Figure** **7**a shows the *operando* XAS measurements of 2dppeCl for light‐off 1 and Figure 7b the measurement for light‐off 2. Similar to the *in situ* measurements, the catalyst initially consists of a mixture of Au_Red_ and Au_Complex_ species. At 290 °C, the Au_Complex_ species starts to convert into Au_Red_ until 440 °C, where simultaneously near‐full CO conversion is achieved. With higher temperatures, Au_Ox_ is formed, until the catalyst is an even mixture of Au_Red_ and Au_Ox_ at 600 °C. During cooling Au_Red_ is formed again, together with minor amounts of Au_Complex_, which also represents the starting point for the next light‐off cycle. During the second light‐off, Au_O_x starts to form again at around 390 °C at almost full conversion.

2POP2 consists, in agreement with the *in situ* XAS measurements under oxidizing and reducing conditions, of almost exclusively Au_Complex_. Again, the conversion into Au_Ox_ at around 235 °C can be witnessed, which steadily increases until 600 °C, where the catalyst consists only of Au_Ox_. Similar to 2dppeCl, the catalyst then forms Au_Red_ during cooling down, which then serves as the starting point for the consecutive light‐off. In line with the laboratory catalytic tests, an improved catalytic activity was observed in the second light‐off, pointing toward the *in* *situ* formation of a stabilizing interaction between remaining phosphorus‐containing species and Au nanoparticles. The formation of Au_Ox_ during the second light‐off reflects the trend observed during the first cycle, starting at 240 °C and slowly increasing with increasing temperature. Au_Ox_ seems to be one of the catalytically active species in both light‐offs, which most likely results from the electronic modulation of Au *via* charge transfer processes with the P‐containing species, resulting from the partial ligand removal. At this point no concise conclusions about any potential movement from ligand molecules or residues from the support to the metal particle can be drawn. However, similar observations were reported by the groups of Bürgi and Barrabés in the past for thiolate‐protected gold clusters.^[^ ^28^ ^]^

POPCl starts similar to the temperature‐programmed reductions (TPR) measurements as fully reduced. However, at 400 °C, after the catalyst starts to become active, it can be observed that slight amounts of Au_Ox_ are formed.

The *operando* measurements indicate, that at higher temperatures and independent of the initial state of the catalyst, Au_Ox_, which represents Au^δ+^ species, is being formed. 2POP2 as the most active catalyst with the smallest Au particle site in the series shows the earliest onset of forming the active Au_Ox_ species. This leads to the assumption that within 2POP2 Au is stabilized by ligand residues in a way that small particles are formed, which are more temperature resistant and show an improved oxidation/reduction behavior, which might be caused by charge transfer processes between the phosphine‐based anchoring points on the support and the metal centers. This Au–P interaction consequently impacts the adsorption and conversion of CO. A similar effect can also be observed for 2dppeCl, whereby the ligands do not affect the Au to a similar degree as 2POP2. 2POPCl further adds to this assumption as TEM imaging shows the largest particles in the initial state for this catalyst. Large particles tend to have a worse oxidation/reduction behavior, which can also be observed in the conducted *operando* experiment, which ultimately leads to a diminished catalytic activity. As previously stated, Ma et al. have shown that when comparing ceria‐supported Pt catalysts, a considerably higher valence of the Pt atoms could be achieved when using P‐doped ceria.^[^ ^55^ ^]^ A similar effect induced by a partial phosphine ligand decomposition and the formation of surface‐stabilized Au nanoparticles could lead to the improved oxidation/reduction behavior that was observed during the *operando* measurements.

**Figure 8:**
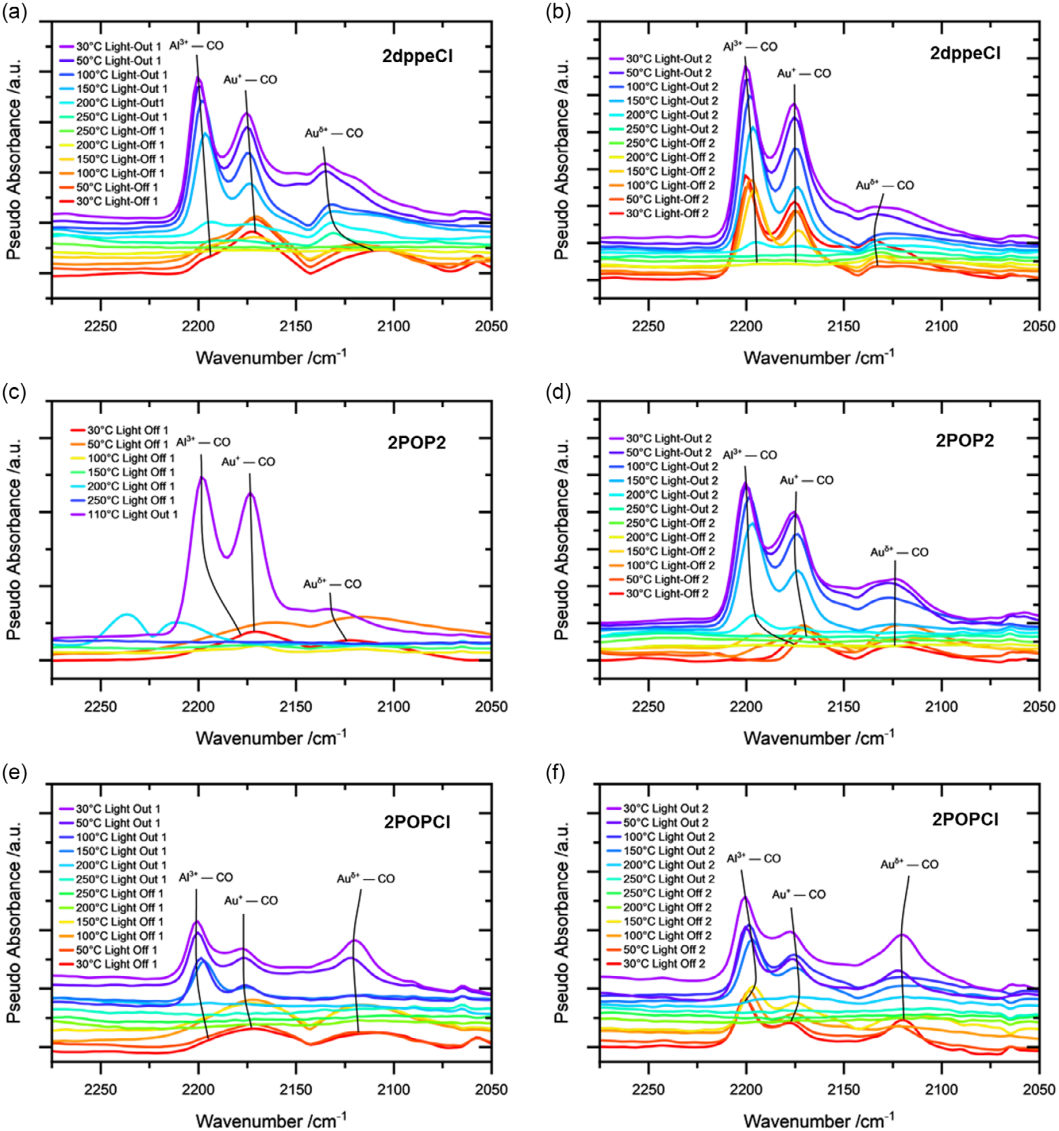
DRIFT spectra of 2dppeCl, 2POP2, and 2POPCl at different temperatures during the light‐off and light‐out of the a,c,e) first catalytic cycle and at different temperatures during the light‐off and light‐out of the b,d,f) second catalytic cycle. IR bands around 2143 cm^−1^ correspond to gas‐phase CO signals. The spectra were converted to pseudoabsorbance according to Meunier et al.^[^ ^64^ ^]^.

Having elucidated the oxidation states exhibited by our Au species during catalysis through *operando* XAS measurements, DRIFTS was employed to investigate the CO adsorption characteristics of the catalysts. By correlating the information obtained from the XAS and DRIFTS analyses, a more comprehensive understanding of the oxidation state and binding species of the Au catalysts is achieved.^[^ ^56^ ^]^ For this, the supported Au complexes were treated with the same reaction mixture used for the CO oxidation catalysis (1000 ppm of CO, 10% O_2_ balanced in N_2_) under different temperatures. Two light‐off and light‐out measurements were conducted while gathering spectral data every 50 °C up to 250 °C. Above 200 °C though, no signals pertaining to CO species could be detected for any of the catalysts, as the desorption of CO to the Au species is preferred to its adsorption. Before letting the catalysts cool down again, the reaction chamber was heated to 350 °C for 30 min to partially mimic the procedure of the CO oxidation in the fixed bed reactor, which was heated to 600 °C between consecutive light‐offs. The DRIFT spectra of 2dppeCl, 2POP2, and 2POPCl at different temperatures during two consecutive light‐off and light‐out cycles are shown in **Figure** **8**.

2dppeCl shows two different CO stretching frequencies at 2195 and 2173 cm^−1^, which can be attributed to Al^3+^–CO and isolated Au^+^–CO species, respectively.^[^ ^57^ ^]^ These bands did not change throughout the first light‐off. During the light‐out at 150 °C and below, an additional signal at ≈2135 cm^−1^, which hints at CO binding to partially oxidized gold species Au^δ+^–CO,^[^ ^57^ ^]^ becomes visible. While the first two signals (Al^3+^–CO and Au^+^–CO) show little‐to‐no difference in wavenumber between the catalysts, the Au^δ+^–CO species at 2135 cm^−1^ is detected at 2130 cm^−1^ for the 2POP2 and 2120 cm^−1^ for the 2POPCl catalyst. During the second light‐off using 2dppeCl as catalyst, as shown in Figure 8b, the three mentioned vibrations are already visible at 30 °C and also remain present during the whole light‐off/out cycle. Thus, the Au sites, generated in the first light‐off, are maintained and serve as the starting point for the second light‐off/light‐out. These observations bolster the fact that the partial ligand removal is essential to enable a stabilizing interaction between the remaining P‐species and the Au nanoparticles, which is necessary to produce active surface‐stabilized catalysts. A similar behavior was observed for the 2POP2 (Figure 8c,d). One distinction though is that during the first light‐off, the contribution of the Au–CO vibrations is very minor. Therefore, the Au‐P binding sites in 2POP2 seem to be almost exclusively formed during the reaction, but are maintained for the following cycles. While the *operando* XAS analysis (Figure 7) showed the variable nature of the oxidation state of the Au species during catalysis, generally depicting an increase in oxidized Au species with increasing temperature and thus CO conversion, DRIFT analyses could further show how the first light‐off is necessary to evolve the catalytically active surface‐stabilized Au species and that this change is maintained for the following light‐offs.

## 3 Conclusion

Various binuclear gold complexes featuring bidentate chelating phosphine ligands, such as dppe and POP, were synthesized and used as molecular precursors for alumina‐supported gold catalysts, which were applied in the oxidation of CO as test reaction. Thorough characterization of these catalysts before, during, and after catalysis provided insights into the influence of ligands on catalyst activity and stability. *Operando* XAS and *in* *situ* DRIFTS revealed that, depending on the choice of ligand in the Au complex, P‐containing ligand residues formed under CO oxidation conditions, leading to charge transfer processes between Au and P.

A key aspect of this study was the stability of the initial molecular complex, which strongly influences the formation of ligand residues on the support and the so‐called surface‐stabilized gold particles. If the complex was overly decomposable, the catalyst exhibited predominantly agglomerated gold species. Conversely, a too stable complex yielded gold nanoparticles with blocked active sites. Both scenarios resulted in lower catalytic activity compared to the best‐performing catalyst derived from the [Au_2_(μ_2_‐POP)_2_]OTf_2_ complex. This complex, which retained stability during impregnation, while allowing for the formation of active gold sites during catalysis, demonstrated enhanced activity and stability compared to the commercially available reference catalyst Au/TiO_2_.

The superior performance of this catalyst is attributed to the formation of phosphorus‐stabilized Au nanoparticles, facilitated by the thermal decomposition of the ligand. The resulting charge transfer between Au and P resulted in the presence of cationic gold species, demonstrating the significant electronic influence of the ligand residue. This is further highlighted by the fact that [Au_2_(μ_2_‐POP)_2_]OTf_2_/Al_2_O_3_ outperforms the P‐free reference catalyst Au/Al_2_O_3_ in terms of catalytic CO oxidation activity. Also, the P‐containing ligand residue increases the sinter resistance of the catalyst. While the study offers valuable insights into the fate of the different Au complexes, future research should also aim to investigate the influence of the phosphine ligands itself on the CO oxidation. This will contribute to a more comprehensive understanding of supported molecular catalysts.

This study underscores the delicate balance between the stabilization of Au particles through the use of ligands and the blocking of active surface sites by strongly coordinating molecules, providing guidelines for using molecular gold complexes as precursors to create highly stable and active surface‐stabilized gold particles.

## 4 Experimental Section

4.1

4.1.1

##### Materials

All experiments were carried out on a Schlenk‐line under Ar atmosphere or in an Ar‐filled glove box (MBraun). Toluene, tetrahydrofuran (THF), *n*‐pentane, and n‐heptane were dried using a MBraun solvent purification system (SPS‐800) and subsequently degassed. THF was additionally distilled from potassium/benzophenone. Acetonitrile (MeCN) and dichloromethane (DCM) were distilled over calcium hydride. Deuterated solvents, such as CDCl_3_ and CD_2_Cl_2_, were dried over P_2_O_5_, while C_6_D_6_, D_8_‐toluene, and CD_3_CN were dried over CaH_8_. Prior to use, deuterated solvents were degassed by three consecutive freeze–pump–thaw cycles. All solvents were stored over activated molecular sieves (MeCN over 3 Å, all other solvents over 4 Å). The compounds tetraphenyldiphosphoxane (POP),^[^ ^43^ ^]^ [Au(tht)Cl] (tht = tetrahydrothiphene)^[^ ^58^ ^]^ [Au(tht)_2_]OTf,^[^ ^58^ ^]^ [Au_2_(μ_2_‐POP)Cl_2_],^[^ ^43^ ^]^ [Au_2_(μ_2_‐POP)_2_]OTf_2_,^[^ ^43^ ^]^ and [Au_2_(μ_2_‐dppe)Cl_2_] (dppe = bis(diphenylphosohinoethane)^[^ ^59^ ^]^ were synthesized via literature known procedures and analyzed using nuclear magnetic resonance (NMR) spectroscopy. The literature known compound [Au_2_(μ_2_‐dppe)_2_]OTf_2_ was synthesized using a slightly modified procedure. For this, 100.0 mg (0.191 mmol, 1.00 eq.) of [Au(tht)_2_]OTf and 76.3 mg (0,191 mmol, 1.00 eq.) of dppe were stirred in 10 mL of DCM for 3 h. Subsequently, the slightly yellow solution was filtered, reduced to half of its volume, and layered with 10 mL of n‐heptane, which resulted in the formation of colorless crystals within 12 h. The crystals were separated from the mother liquor, washed with n‐pentane, and dried under vacuum.

All chemicals, beside solvents, were used without any further purification. Puralox catalox 100/150 Al_2_O_3_ from Sasol was used as support material, which was calcined at 700 °C for 5 h prior to use. Abcr supplied tetrahydrothiphen (98%), diphenylphonphineoxide (97%), and triethylamine (99%). Chlorodiphenylphosphine (>97%) and 1,2‐bis(diphenylphosphino)ethane (>97%) were purchased from TCI Chemicals and silver triflate (99%) was bought from Sigma Aldrich. The Au/TiO_2_ catalyst was purchased from abcr.

##### Catalyst Preparation

The preparation of the catalysts took place under air with the use of nondried solvents. The stoichiometric amounts were selected according to a gold loading of 0.2 wt%. All catalysts were used without any further thermal treatment.

##### Catalyst Preparation: [Au_2_(μ_2_‐POP)_2_]OTf_2_/Al_2_O_3_ (2POP2)

7.4 mg of [Au_2_(μ_2_‐POP)_2_]OTf_2_ were dissolved in 2 mL acetone and added in 1 mL portions to 1.00 g of Al_2_O_3_ support. The powder was dried between the complex additions for 5 min at 60 °C. After the complete addition of the metal precursor solution, the sample was further dried for 6 h at 60 °C.

##### Catalyst Preparation: [Au_2_(μ_2_‐POP)Cl_2_]/Al_2_O_3_ (2POPCl)

The same method as mentioned above was used, except that 4.3 mg of [Au_2_Cl_2_(μ_2_‐POP)_2_Cl_2_] were dissolved in THF.

##### Catalyst Preparation: [Au_2_(μ_2_‐dppe)_2_]OTf_2_/Al_2_O_3_ (2dppe2)

The same method as mentioned above was used, except that 7.6 mg of [Au_2_(μ_2_‐dppe)_2_]OTf_2_ were dissolved in DCM.

##### Catalyst Preparation: [Au_2_(μ_2_‐dppe)Cl_2_]/Al_2_O_3_ (2dppeCl)

The same method as mentioned above was used, except that 4.4 mg of [Au_2_(μ_2_‐dppe)_2_Cl_2_] were dissolved in THF upon slight heating (50 °C).

##### Catalyst Preparation: Au/Al_2_O_3_ (AuRef)

The same method as mentioned above was used, except that 4.0 mg of H[AuCl_4_]·3H_2_O were dissolved in water and the drying time between portions was increased to 30 min. The sample was obtained as a light purple powder.

##### Catalytic testing: Light‐Off Experiments

The catalyst bed was prepared by diluting the catalyst sieved fraction (grain size of 125–250 μm) with SiO_2_ (grain size of 125–250 μm) to yield a total mass of 1.00 g and to minimize effects by heat and mass transfer. For each experiment, the amount of Au catalyst was kept constant at 200 mg and the gas flow was adjusted accordingly, resulting in a constant WHSV of 60 000 L gAu^−1^ h^−1^. The catalysts were placed in quartz tubes (outer diameter: 10 mm; inner diameter: 8 mm) and fixed with two quartz wool plugs. Two thermocouples inside the reactor were positioned at the start and end of the catalyst bed to monitor the temperature during the reaction. The reaction gas mixture was dosed by mass flow controllers (Bronkhorst) containing 1000 ppm of CO, 10% O_2_ balanced in N_2_. The gas composition was monitored at the reactor outlet by on‐line infrared spectroscopy (FTIR with gas cell, MKS Instruments). The catalytic activity was monitored in the range of 50–600 °C with a heating ramp of 5 K min^−1^. After the light‐off, the catalyst was kept at 600 °C for 10 min and was then cooled down to 50 °C with a heating ramp of 5 K min^−1^. The whole test cycle consisted of two such consecutive light‐offs for each material.

##### Analytical Methods: Nuclear Magnetic Resonance (NMR)

NMR spectra were recorded on a Bruker AVANCE III or a Bruker AVANCE Neo 400 MHz spectrometer at 298 K. Chemical shifts are given in ppm and are referenced to the residual solvent signals of the deuterated solvents. Unambiguous assignments were made on the basis of chemical shifts, coupling patterns, and 2D NMR experiments (^1^H−^1^H COSY, ^1^H−^13^C HMQC, and ^1^H−^13^C HMBC).

##### Analytical Methods: Transmission Electron Microscopy (TEM)

For the TEM measurements, the as‐prepared and spent catalyst powders were first dispersed in water and then casted on to a copper grid with lacey carbon on top. The support microstructures and Au particle size distribution were characterized by high‐angle‐annular dark‐field scanning transmission electron microscopy (HAADF‐STEM) in a FEI Titan 80–300 microscopy operated at 200 kV. The Au particle size distribution was determined on HAADF‐STEM images by using the Fiji software.^[^ ^60^ ^]^

##### Analytical Methods: Operando X‐ray Absorption (XAS) Spectroscopy

XAS measurements were performed at the P65 beamline^[^ ^61^ ^]^ at DESY (Hamburg, Germany). The experiments were performed in a quartz capillary microreactor (1.5 mm outer diameter, 0.01 mm wall thickness) loaded with 6 mg of granulated Au/Al_2_O_3_ catalyst under the same reaction conditions described earlier, also in accordance with previous studies,^[^ ^62^ ^]^ with the exemption of a higher WHSV of 170 000 L gAu^−1^ h^−1^ and He as a carrier gas. Additionally to the light‐off experiments, TPR with 1000 ppm CO in He and temperature‐programmed oxidation (TPO) with 10 vol% O_2_ in He were performed until a temperature of 600 °C with a heating ramp of 5 K min^−1^. Before and after the transient experiments, extended X‐ray absorption fine structure spectra (EXAFS) were taken in the energy range from 11 770 to 12 920 eV using a total acquisition time of 5 min. The EXAFS measurements were repeated 3 times and averaged for the data evaluation. During the heating and cooling phase of the experiments X‐ray absorption near‐edge structure (XANES) spectra at the middle position of the catalyst bed were recorded. Here, the acquisition time for each spectrum was shortened to 2 min with an energy resolution of 12 370 eV. One XANES spectra was collected every two minutes and to improve the data quality three spectra were merged resulting in one merged spectrum each 6 min or 30 K. The measurements were performed in fluorescence mode using a silicon drift detector (Vortex‐90EX X‐ray detector). The energy of the incident X‐ray beam was tuned by a double‐crystal monochromator with Si(111) crystals. The slice of the beam was adjusted to 1 × 1 mm^2^ by using slits. Since the Au samples were very prone to beam damage, attenuators (5 mm of glassy carbon) were inserted into the beam before the monochromator and additional 3 mm of aluminum foil were inserted into the beam before the reactor. This led to a low signal‐to‐noise ratio and also hindered the EXAFS analysis.

##### Analytical Methods: Thermogravimetric analysis (TG)

Simultaneous TG‐MS was performed with a STA 449 F3 Jupiterand a QMS 403 Aëolos (Netzsch) using quartz glass crucibles. The TG‐MS experiments were performed under oxidizing atmosphere containing 10 vol% O_2_ in Ar. 25 mg of the as‐prepared powders were filled into the crucible. The setup was flushed at ambient temperature with the gas mixture. The measurements followed the sequence: 2 h at 30 °C, heating to 600 °C with 5 C min^−1^, and then cooling to room temperature.

##### Analytical Methods: Powder X‐ray Diffraction (PXRD)

PXRD measurements were performed using a Stoe STADI‐MP diffractometer operating with Ge‐monochromatized Cu (*λ* = 1.54178 Å) in transmission mode. The Scherer equation was used to determine the gold crystallite sizes based on the reflexes at 38.1°, 44.3°, and 77.5° using a Scherer constant of 0.94.^[^ ^63^ ^]^ The full width half maximum was identified using the Match! phase analysis software. In addition to the freshly prepared state, the samples underwent two separate thermal treatments at 400 and 600 °C in air for 10 min prior to measurement.

##### Analytical Methods: Attenuated Total Reflection Infrared Spectroscopy (ATR‐IR)

ATR‐IR measurements were carried out in the region of 4000–400 cm^−1^ using a Bruker Tensor 37 FTIR spectrometer equipped with a room‐temperature DLaTGS detector, a diamond ATR unit, and a nitrogen‐flushed measurement chamber. For one measurement, the samples were pretreated at 600 °C in air for 10 min.

##### Analytical Methods: Inductively Coupled Plasma Optical Emission Spectroscopy (ICP‐OES)

The gold loading of the catalysts was determined with ICP–OES which was done at the Institute for Applied Materials at the Karlsruhe Institute of Technology using the iCAP7600 DUO from Thermo‐Fisher‐Scientific. Prior to analysis, the samples were digested using acids and high pressure. Each measurement was repeated twice.

##### Analytical Methods: Diffuse Reflectance Infrared Fourier Transform Spectroscopy (DRIFTS)

DRIFTS measurements were performed in a Bruker Vertex 70 instrument with a nitrogen‐cooled mercury cadmium telluride detector and a Praying Mantis high‐temperature reaction chamber equipped with a CaF_2_ window. The temperature inside the cell was controlled with two heating cartridges and a water‐cooling system. The catalyst bed was prepared by filling the sample holder first with CaF_2_ powder. On top of the CaF_2_ powder, a thin layer of ground catalyst (≈25 mg, sieve fraction 125–250 μm) was distributed. The gas stream in to the cell was kept constant at 100 mL min^−1^. The sample surface was cleaned under Ar for 1 h at 250 °C, while cooling down, a background spectrum was taken at every temperature step, spectra were recorded (30 scans, 4 cm^−1^ resolution). The gas composition was then switched to 1000 ppm CO/Ar and spectra were recorded. The spectra were converted to pseudoabsorbance according to Meunier et al.^[^ ^64^ ^]^

##### Analytical Methods: Surface Area Measurements

The specific surface area was measured by N_2_ physisorption using the Brunauer–Emmett–Teller (BET) method.^[^ ^65^ ^]^ The measurements were carried out with a AUTOSORB IQ‐XR VITON (Anton Paar). The samples were treated at 150 °C for 3 h under reduced pressure prior to the measurement.

## Conflict of Interest

The authors declare no conﬂict of interest.

## Author Contributions

**Fabian Rang**: Investigation (lead); Writing—original draft (equal). **Tim Delrieux**: Methodology (lead); Writing—original draft (equal). **Florian Maurer**: Conceptualization (equal); Formal analysis (equal). **Franziska Flecken**: Data curation (supporting). Investigation (supporting). **Jan‐Dierk Grunwaldt**: Conceptualization (equal). **Schirin Hanf**: Writing—original draft (lead); Writing—review and editing (lead). **Fabian Rang** and **Tim Delrieux** contributed equally to this work.

## Supporting information

Supplementary Material

## Data Availability

The data that support the findings of this study are openly available in Zenodo at DOI: 10.5281/zenodo.11 941 122, reference number.^[^ ^66^ ^]^
