# Supplementary material for: Supported Binuclear Gold Phosphine Complexes as CO Oxidation Catalysts: Insights into the Formation of Surface‐Stabilized Au Particles
Source: Small Sci. 2024 Oct 14;4(12):2400345. doi: 10.1002/smsc.202400345 (PMC11935119; doi:10.1002/smsc.202400345)
Supplement: Supplementary file 1 — Supplementary Material [file SMSC-4-2400345-s001.pdf]

Supporting Information

Supported binuclear gold phosphine complexes as CO oxidation catalysts: insights into the formation of surface-stabilized Au particles

Fabian Rang,[a]‡ Tim Delrieux,[b]‡ Florian Maurer,[b] Franziska Flecken,[a] Jan-Dierk Grunwaldt[b,c] and Schirin Hanf[a]*

[a] Institute for Inorganic Chemistry, Karlsruhe Institute of Technology, Engesserstr. 15, 76131 Karlsruhe, Germany.

[b] Institute for Chemical Technology and Polymer Chemistry, Karlsruhe Institute of Technology, Engesserstr. 18 / 20, 76131 Karlsruhe, Germany.

[c] Institute of Catalysis Research and Technology, Hermann-von-Helmholtz-Platz 1, 76344 Eggenstein-Leopoldshafen, Germany.

‡ These authors contributed equally to this work.

Raw data concerning this publication was deposited on Zenodo (DOI: 10.5281/zenodo.11941122).

1. Catalyst characterization

ATR-IR spectroscopy

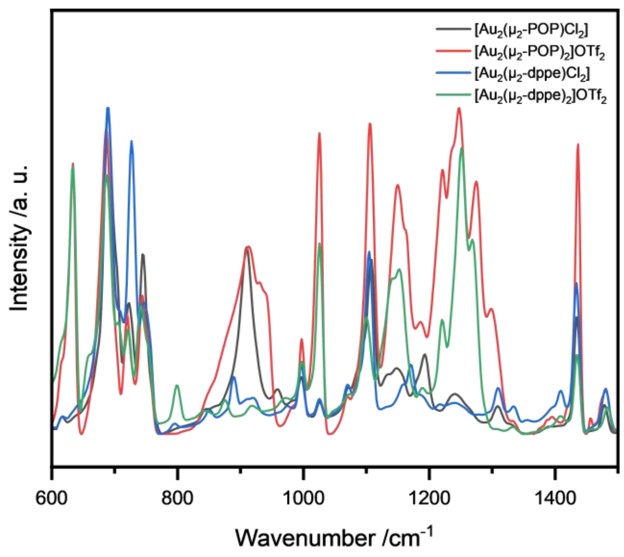

Figure S1: ATR-IR spectra of the molecular gold phosphine complexes.

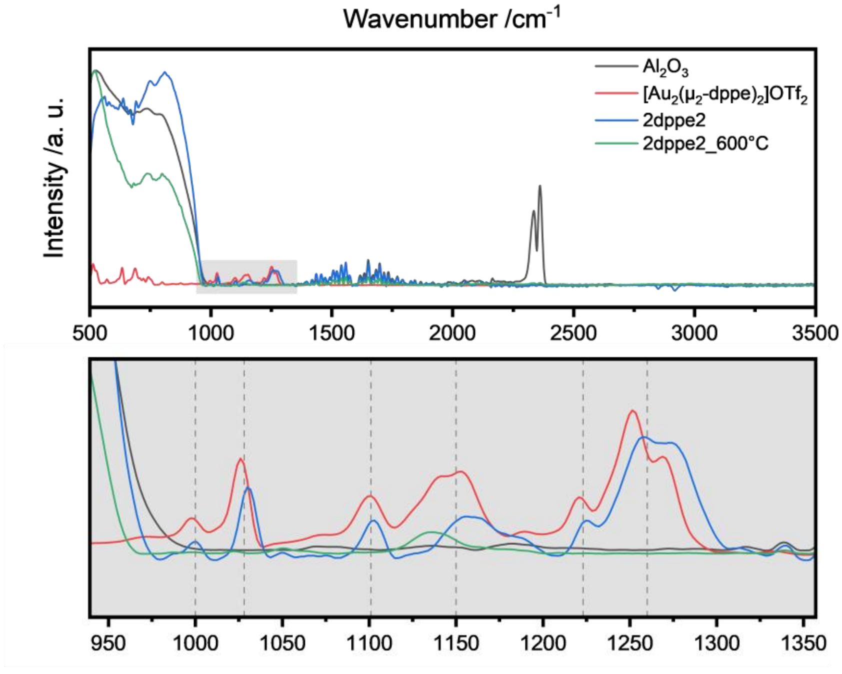

Figure S2: ATR-IR spectra of **2dppe2**. Black: Al_2O_3 support, red: gold complex, blue: impregnated catalyst, green: catalyst treated at 600 °C for 10 min. Dashed lines indicate strong vibrations of the complex.

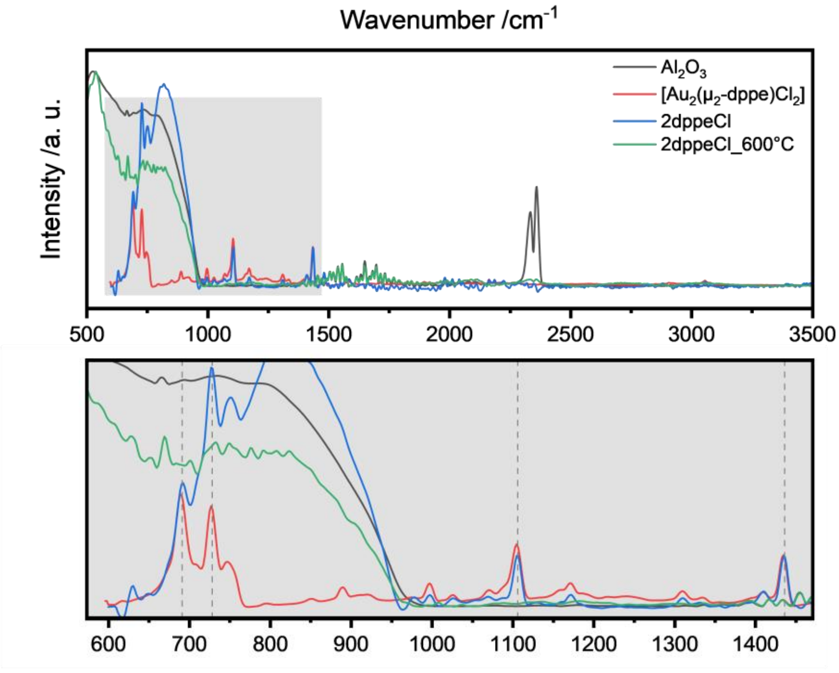

Figure S3: ATR-IR spectra of **2dppeCl**. Black: Al_2O_3 support, red: gold complex, blue: impregnated catalyst, green: catalyst treated at 600 °C for 10 min. Dashed lines indicate strong vibrations of the complex.

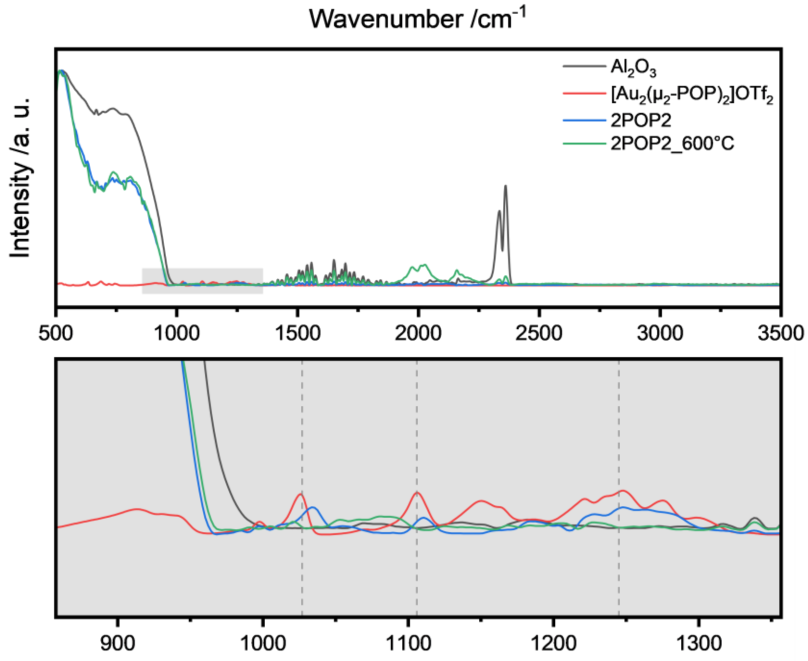

Figure S4: ATR-IR spectra of **2POP2**. Black: Al_2O_3 support, red: gold complex, blue: impregnated catalyst, green: catalyst treated at 600 °C for 10 min. Dashed lines indicate strong vibrations of the complex.

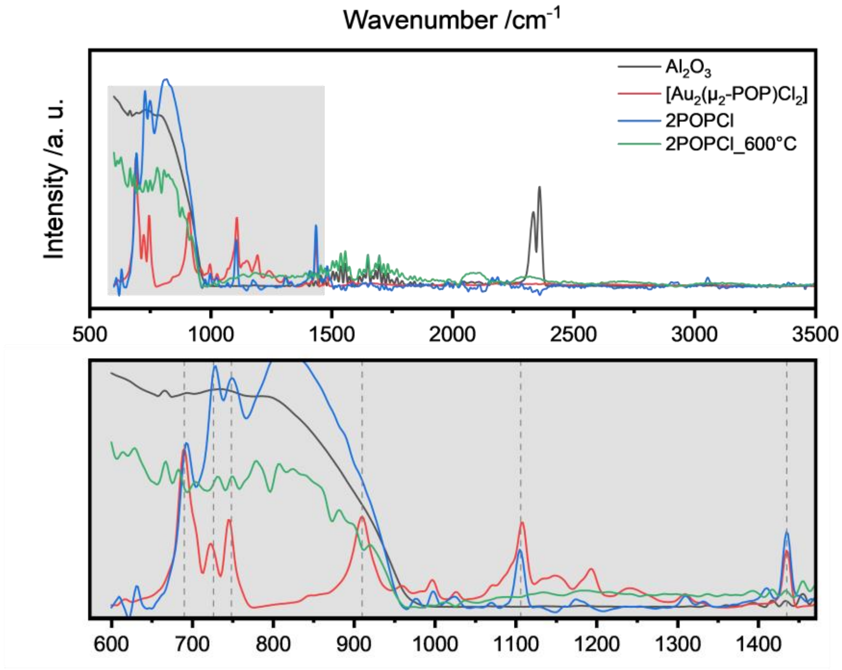

Figure S5: ATR-IR spectra of **2POPCl**. Black: Al_2O_3 support, red: gold complex, blue: impregnated catalyst, green: catalyst treated at 600 °C for 10 min. Dashed lines indicate strong vibrations of the complex.

Decomposition temperature measurements

Table S1: Decomposition temperatures of the dinuclear gold complexes.

	$[Au_2(\mu_2\text{-POP})Cl_2]$	$[Au_2(\mu_2\text{-POP})_2]OTf_2$	$[Au_2(\mu_2\text{-dppe})Cl_2]$	$[Au_2(\mu_2\text{-dppe})_2]OTf_2$
Decomposition Temperature	115-125°C	150-170°C	260-280°C	260-280°C

ICP-OES measurements and specific surface area

Table S2: ICP-OES and BET measurement results.

	2dppeCl	2dppe2	2POPCl	2POP2	AuRef	Au/TiO_2	Al_2O_3
Gold loading / wt% (ICP-OES)	0.218	0.182	0.258	0.185	0.131		
Surface area / m^2/g (BET)	110	88	110	91	111	90	107

Thermogravimetric analysis coupled with mass spectrometry

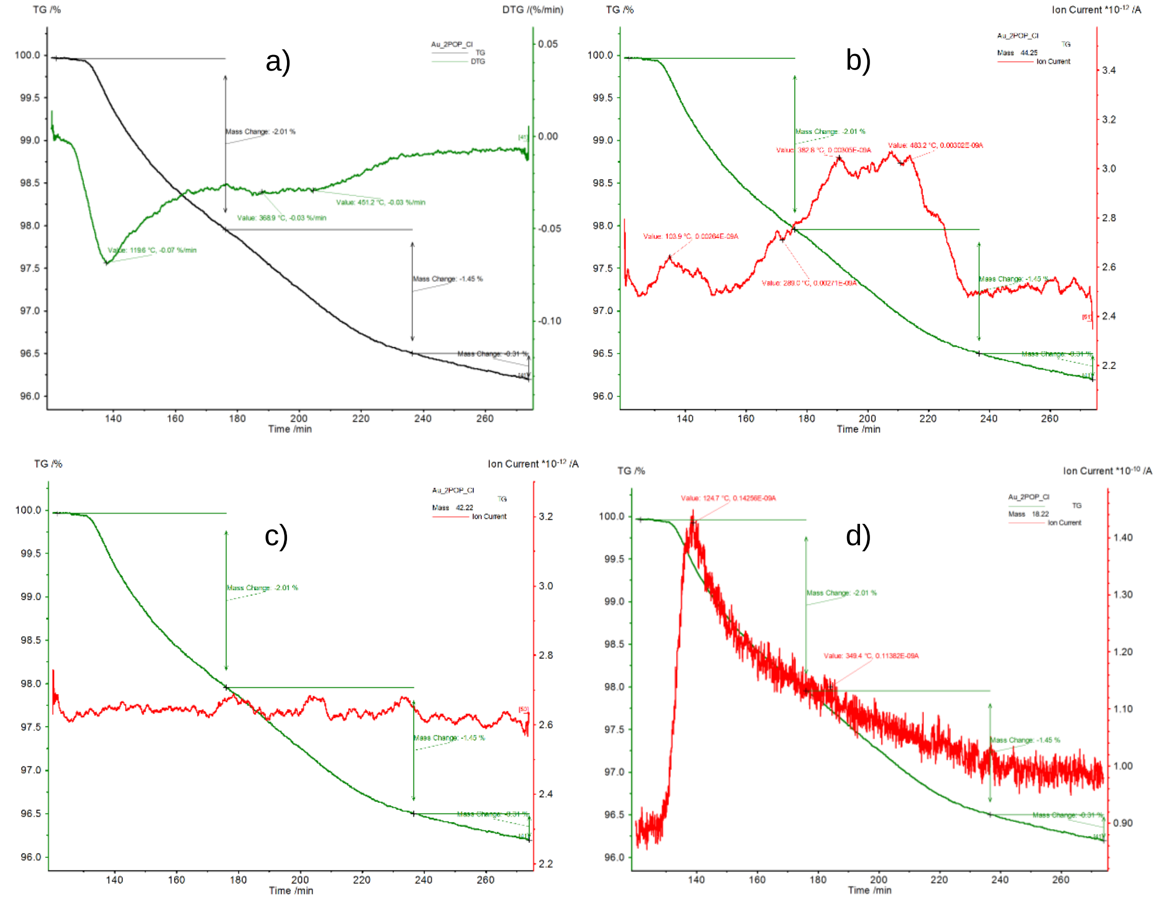

Figure S6: a) TG-DTA graph of **2POPCl**. b) Graph of TGA with MS of ion mass 44 (CO_2^{n+}). c) Graph of TGA with MS of ion mass 42 ($C_3H_6^{n+}$). d) Graph of TGA with MS of ion mass 18 (H_2O^{n+}).

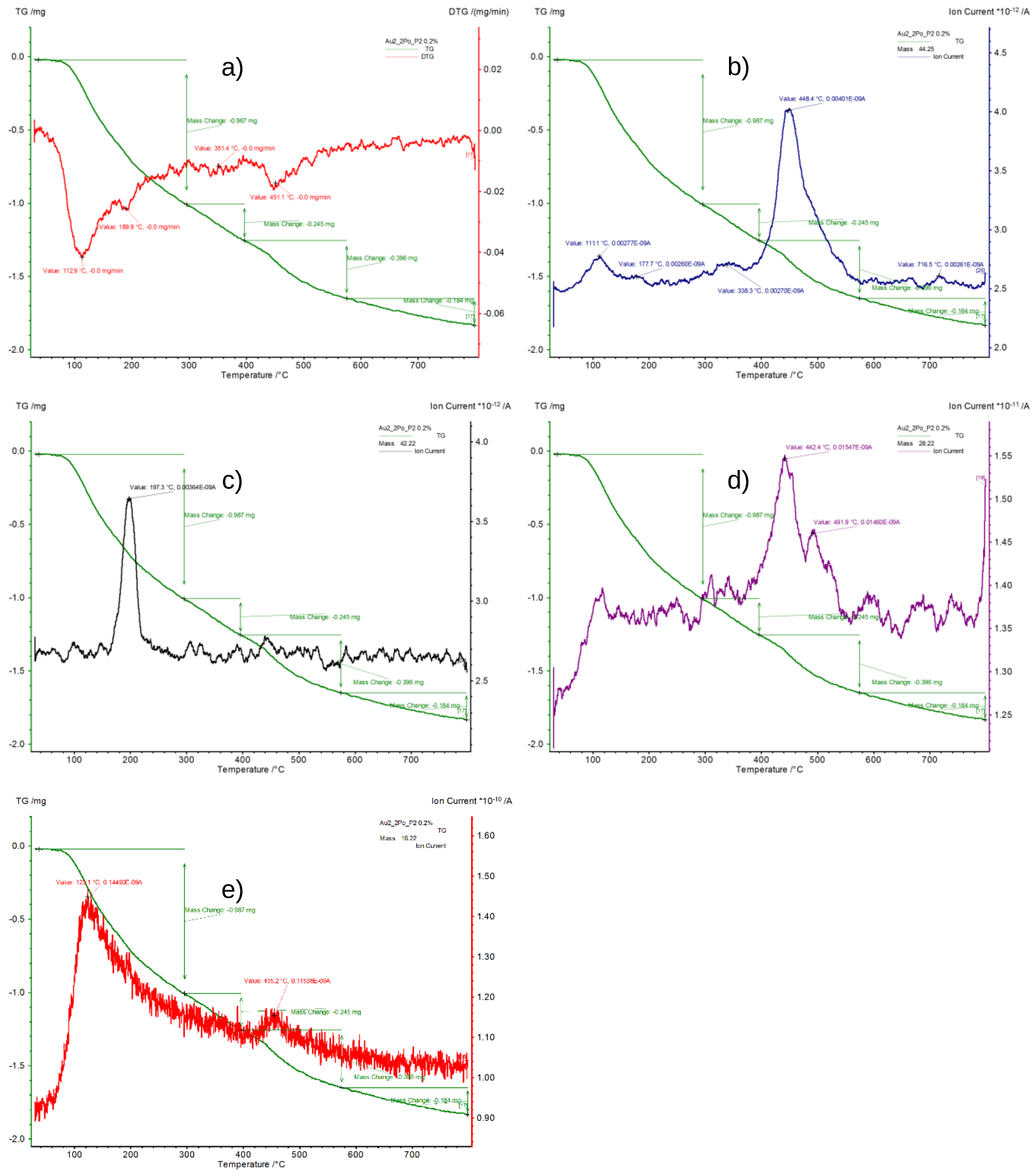

Figure S7: a) TG-DTA graph of **2POP2**. b) Graph of TGA with MS of ion mass 44 (CO_2^{n+}). c) Graph of TGA with MS of ion mass 42 ($C_3H_6^{n+}$). d) Graph of TGA with MS of ion mass 28 (CO^{n+}). e) Graph of TGA with MS of ion mass 18 (H_2O^{n+}).

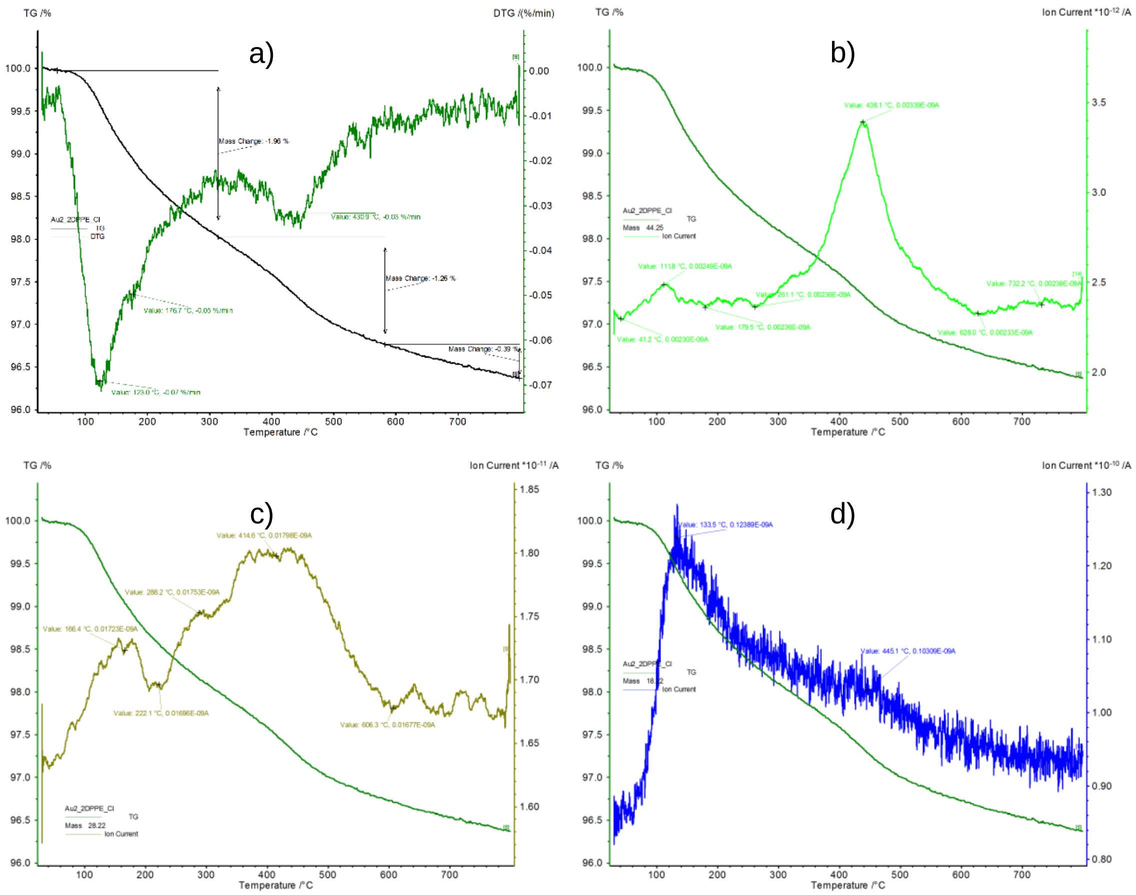

Figure S8: a) TG-DTA graph of **2dppeCl**. b) Graph of TGA with MS of ion mass 44 (CO_2^{n+}). c) Graph of TGA with MS of ion mass 28 (CO^{n+}). d) Graph of TGA with MS of ion mass 18 (H_2O^{n+}).

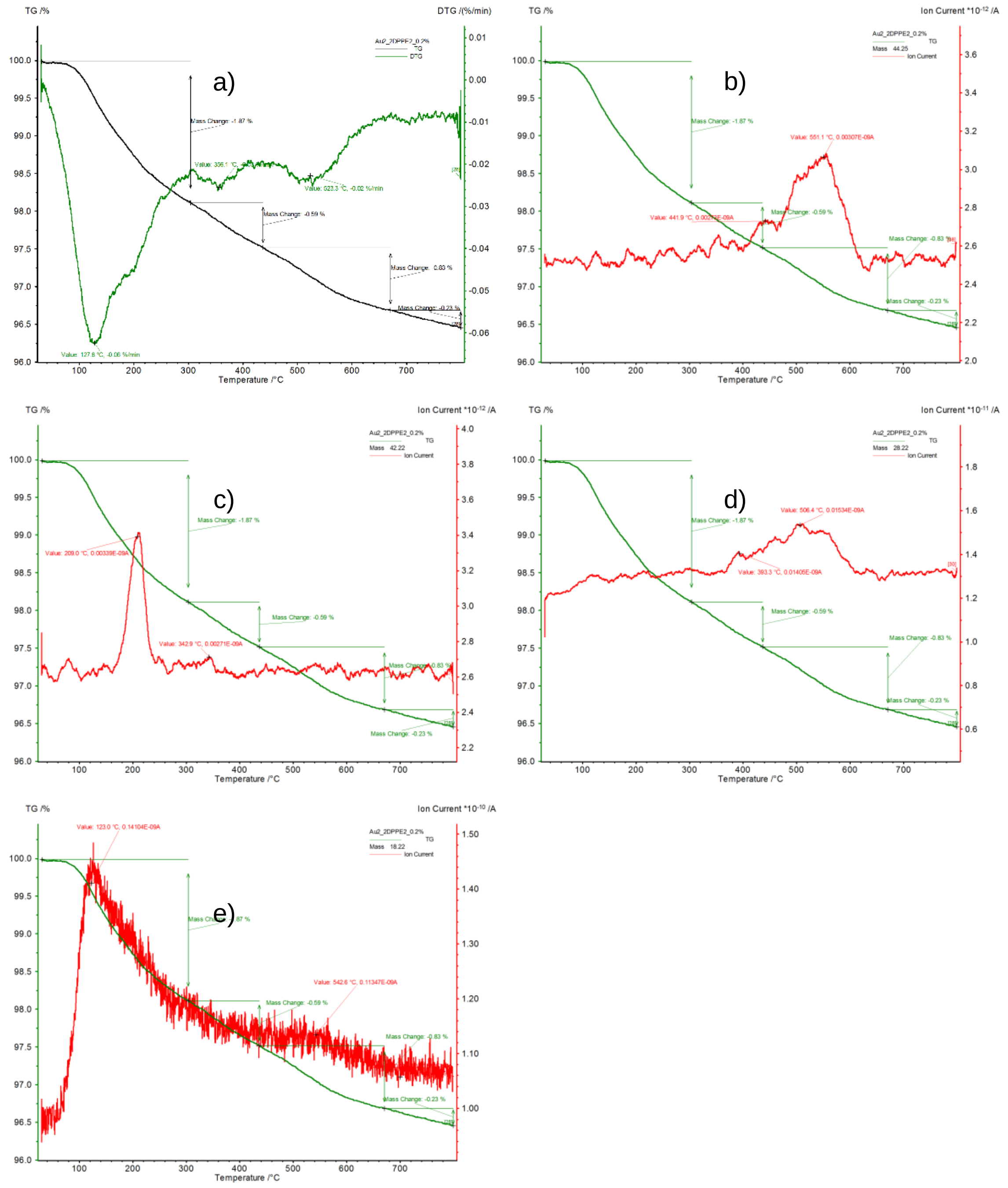

Figure S9: a) TG-DTA graph of **2dppe2**. b) Graph of TGA with MS of ion mass 44 (CO_2^{n+}). c) Graph of TGA with MS of ion mass 42 ($C_3H_6^{n+}$). d) Graph of TGA with MS of ion mass 28 (CO^{n+}). e) Graph of TGA with MS of ion mass 18 (H_2O^{n+}).

2. Catalyst testing

Table S3: Reaction rates for the second LO at the highest recorded CO conversion. Measurements were conducted using a gas mixture of 1000 ppm CO, 10 vol.% O_2 in N_2.

	**2dppeCl**	**2dppe2**	**2POPCl**	**2POP2**	**AuRef**	**Au/TiO$_2$**
**Rate / $mol_{CO} \cdot mol_{Au}^{-1} \cdot s^{-1}$**	0.057	0.058	0.05	0.0670	0.085	0.057
**Temperature / °C**	320	600	500	300	600	390

Table S4: Reaction rate of literature reported Au catalysts on different supports. Determined at 300 K, 20 mL/min, 100 kPa total pressure, 3.3 kPa CO, 1.7 kPa O_2 in He.[1]

	**Au/Al$_2$O$_3$**	**Au/TiO$_2$**	**Au/SiO$_2$**
**Rate / $mol_{CO} \cdot mol_{Au}^{-1} \cdot s^{-1}$**	0.46	0.16	0.016
**Gold loading / wt%**	0.30	0.63	1.0

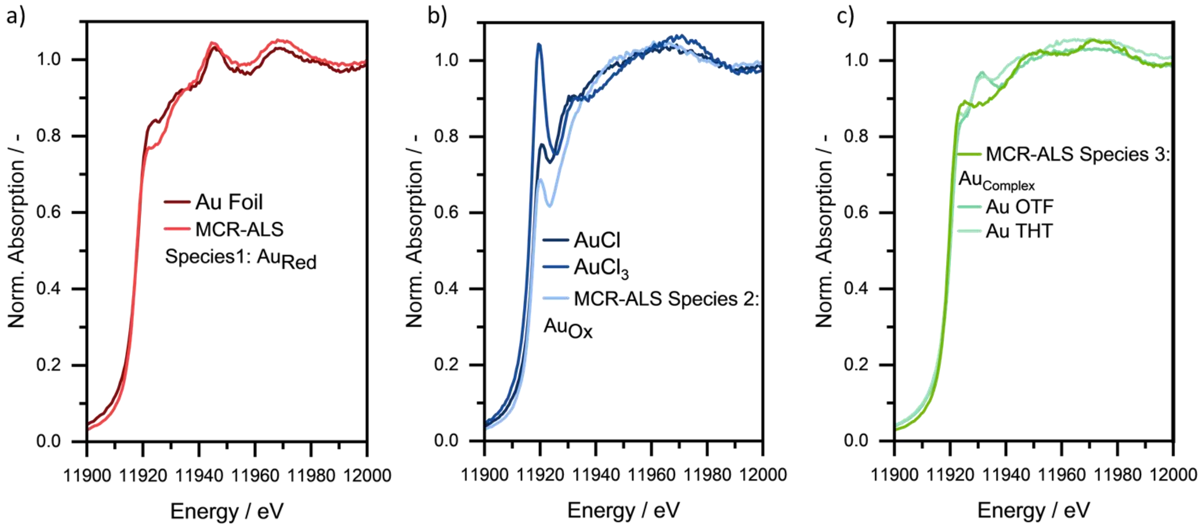

Figure S10: Assigned reference spectra at the Au L3-edge derived from MCR-ALS with the corresponding Au references referring to a) Au_{red} and metallic Au, b) Au_{Ox}, AuCl and $AuCl_3$ and c) $Au_{complex}$ and [Au(tht)Cl] (Au THT) and $[Au(tht)_2]$OTf (Au OTF).

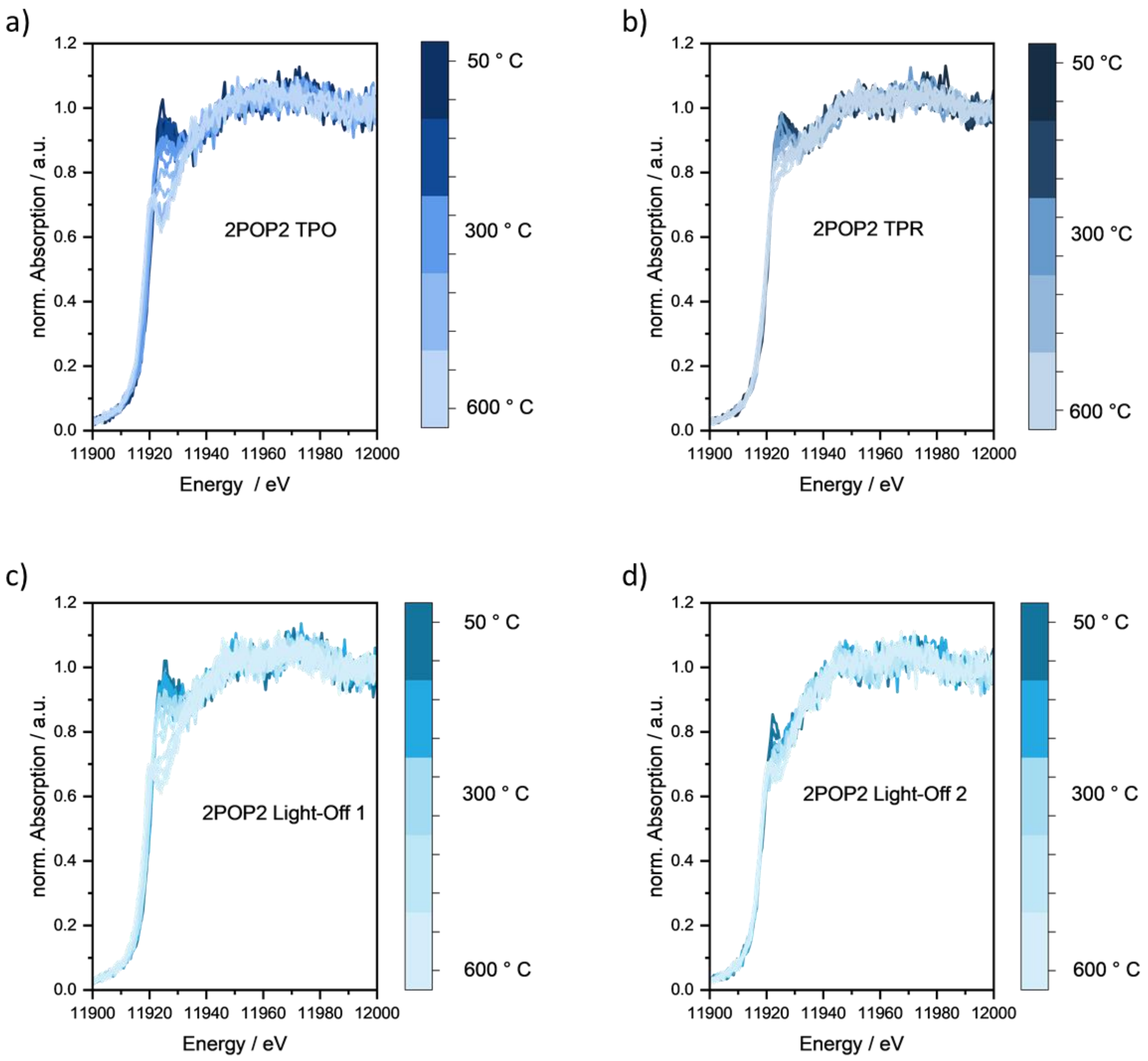

Figure S11: Normalized *in-situ* (a and b) and *operando* (c and d) XANES spectra of **2POP2** recorded at the Au L3-edge.

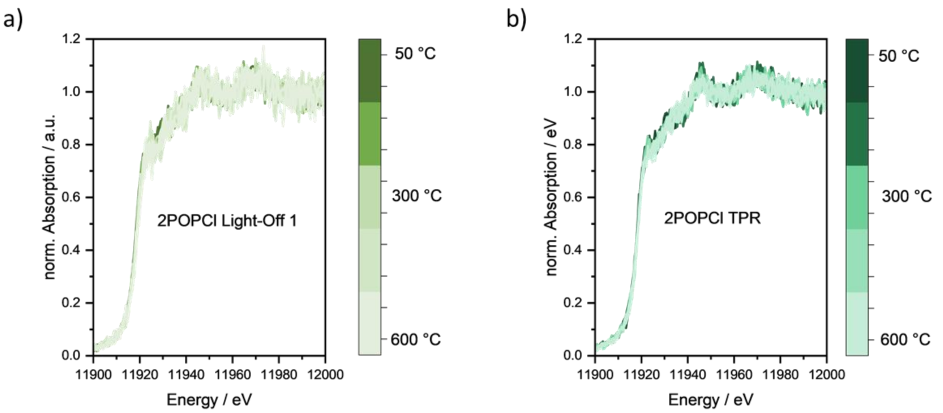

Figure S12: Normalized *operando* (a) and *in-situ* (b) XANES spectra of **2POPCl** recorded at the Au L3-edge.

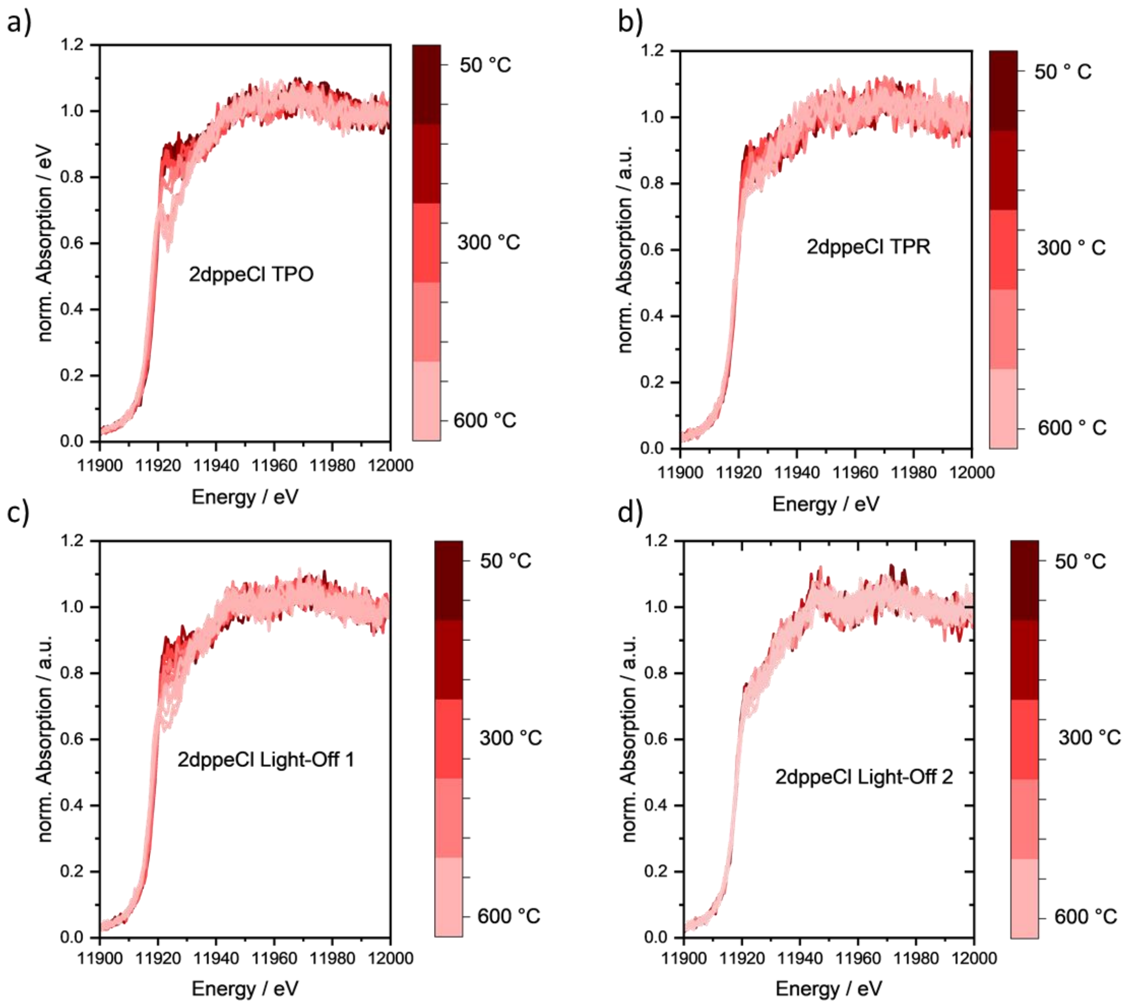

Figure S13: Normalized *in-situ* (a and b) and *operando* (c and d) XANES spectra of **2dppeCl** recorded at the Au L3-edge.

3. References

(1) Weiher, N.; Bus, E.; Delannoy, L.; Louis, C.; Ramaker, D. E.; Miller, J. T.; van Bokhoven, J. A. Structure and Oxidation State of Gold on Different Supports under Various CO Oxidation Conditions. *J. Catal.* **2006**, *240*, 100–107. https://doi.org/10.1016/j.jcat.2006.03.010.
